# Role of mitochondria-associated ER membranes in lipid metabolism: implications for renal lipotoxicity

**DOI:** 10.1042/BSR20260413

**Published:** 2026-09-04

**Authors:** Yile Gao, Minghui Shao, Ya Fang, Jiangping Zeng, Hong Sun

**Affiliations:** Department of Endocrinology and Metabolism, Suzhou Dushu Lake Hospital, The Fourth Affiliated Hospital of Soochow University, Medical Center of Soochow University, Suzhou, Jiangsu 215123, China

**Keywords:** ELD, kidney, lipotoxicity, MAM, mitochondria dysfunction, oxidative stress

## Abstract

Dysregulated lipid metabolism is implicated in renal injury associated with diabetic nephropathy, acute kidney injury, chronic kidney disease, nephrotic syndrome, and renal cell carcinoma. However, its causal role and mechanisms remain ambiguous. Mitochondria-associated ER membranes (MAMs) are contact sites between the endoplasmic reticulum and mitochondria that facilitate the integration of lipid trafficking, mitochondrial metabolism, calcium signaling, and redox homeostasis within cells. Recent evidence from patient biopsies and experimental renal models suggests that altered MAM integrity is linked to ectopic lipid deposition, mitochondrial dysfunction, oxidative stress, and renal injury. The present review examines evidence suggesting that MAM dysregulation may contribute to the abnormal metabolism of phospholipids (PLs), ceramides, cholesterol, fatty acids, and triglycerides in renal cells, thereby addressing a gap between previous reviews on renal lipotoxicity and those focusing on MAM-dependent calcium signaling in kidney diseases. Key mechanisms include impaired PL transfer with disrupted cardiolipin remodeling, ceramide-associated mitochondrial injury, defective fatty acid oxidation, and acyl-CoA synthetase long-chain family member 4-mediated PL peroxidation, leading to renal ferroptosis. Direct evidence for MAM-regulated lipid droplet degradation in the kidney is limited; thus, findings from non-renal cells are differentiated from kidney-specific observations. MAM-associated proteins have emerged as potential therapeutic targets in preclinical studies. However, renoprotective effects of sodium-glucose cotransporter 2 inhibitors and glucagon-like peptide-1 receptor agonists related to MAMs remain indirect and necessitate validation. Restoring the structural and functional integrity of MAMs could represent a promising strategy to mitigate lipid-induced renal injury.

## Introduction

Disordered lipid metabolism is increasingly recognized as a key driver of progressive kidney disease of multiple etiologies, including diabetic nephropathy (DN), acute kidney injury (AKI), and chronic kidney disease (CKD) [1]. Ectopic lipid deposition (ELD) is a pathological hallmark of this process, defined as the excessive accumulation of lipids in non-adipose tissues, and is termed fatty kidney when occurring in the renal parenchyma [2,3]. ELD has been documented in a wide spectrum of kidney diseases, including AKI, DN, CKD, nephrotic syndrome (NS), and renal cell carcinoma (RCC), as summarized in recent comprehensive reviews [2]. The pathogenic significance of renal lipid accumulation was first highlighted in 1982 when Moorhead proposed the lipid nephrotoxicity hypothesis, suggesting that lipid overload accelerates renal dysfunction and disease progression [4]. This initial observational framework has since been expanded and validated to establish a well-recognized paradigm of renal lipotoxicity [5]. The renal lipotoxicity paradigm holds that ectopic lipid accumulation in renal parenchymal cells is not only a morphological alteration but also triggers downstream pathogenic cascades, including oxidative stress, endoplasmic reticulum stress (ERS), and inflammation [6]. These processes ultimately lead to structural and functional renal damage [7,8]. A central feature of this process is dysregulated intracellular lipid handling within renal cells [2]; however, its precise mechanisms remain unclear.

The endoplasmic reticulum (ER) and mitochondria are the two core organelles governing cellular lipid synthesis, trafficking, and catabolism, with their functional coupling primarily mediated by the mitochondria-associated ER membranes (MAMs) [9]. While early studies largely focused on the independent functions of the ER and mitochondria, recent research has shifted to their structural interactions and bidirectional functional crosstalk [9]. This interplay is mediated by specialized tethering sites, where the outer membrane of the ER and the outer mitochondrial membrane (OMM) are positioned in tight apposition to the ER through specific protein-protein interactions. These contact sites were originally defined as MAMs in the seminal work by Vance in 1990 [10]. As specialized lipid raft-like microdomains, MAMs are enriched with over 1000 proteins that integrate lipid metabolism, calcium signaling, ERS responses, inflammation, and autophagy, establishing them as a central regulatory platform for cellular homeostasis [11–13]. In mammalian cells, the ER-mitochondria distance varies by cell type and is approximately 5–20 nm in renal tubular epithelial cells [14]. This specific intermembrane spacing is essential for accurate and efficient inter-organelle communication. Both excessive and insufficient contacts can perturb this structural organization, leading to MAM dysfunction [15,16].

Increasing attention has been directed toward the involvement of MAM in the regulation of intracellular lipids. In diet-induced obese mice, Beaulant et al. demonstrated that the disruption of ER-mitochondria interactions is an early event preceding hepatic insulin resistance and steatosis. Moreover, hepatic overexpression of the organelle spacer FATE1 (fetal and adult testis expressed 1) was sufficient to disrupt ER-mitochondria interactions and induce hepatic steatosis, whereas reinforcement of these contacts through a synthetic linker prevented diet-induced glucose intolerance after 4 weeks of overnutrition [16]. Similar trends were observed in the kidneys. Yang et al. demonstrated a progressive, stage-dependent decline in MAM integrity in renal biopsies from patients with DN, which correlated with increased lipid accumulation and deteriorating kidney function [17]. However, the specific mechanism by which MAM dysfunction drives lipotoxicity, particularly in renal cells, remains unclear.

The present review expands on the existing knowledge of MAM function in lipid metabolism and examines the pathological implications of lipid dysregulation in kidney diseases. It emphasizes MAM-dependent modifications in the metabolism of key lipid classes, such as phospholipids (PLs), sphingolipids, cholesterol (CHOL), fatty acids (FAs), and triglycerides (TGs). Additionally, it addresses lipid droplet (LD) homeostasis, which is a cellular lipid-storage mechanism rather than a separate lipid class. However, these lipid alterations are not uniformly pathogenic. The renal consequences of lipid dysregulation depend on the specific lipid species affected, its subcellular distribution, the surrounding metabolic context, and the extent and duration of dysregulation. Therefore, we discuss how MAM dysfunction may contribute to ELD and lipotoxic injury in renal cells, while distinguishing direct kidney-specific evidence from observations derived from non-renal systems. We also discuss how MAM dysfunction may enhance oxidative stress and lipid peroxidation, thereby amplifying lipotoxic stress. However, renal injury is multifactorial, and non-lipid mechanisms, including hemodynamic abnormalities, inflammation, hyperglycemia, ischemia, and nephrotoxic insults, may independently initiate or aggravate renal damage. Finally, we underscore the therapeutic potential of targeting MAM to support the development of renoprotective strategies and clarify the mechanisms underlying existing interventions.

## MAM dysfunction drives renal ELD and injury

As early as the 1990s, Vance et al. identified a prominent enrichment of lipid metabolism in the MAM [10,18]. According to current research, MAM directly participates in lipid metabolism through three major mechanisms: (1) Lipid synthesis: Numerous studies have shown that MAM is enriched with various lipid-synthesizing enzymes [13]. (2) Lipid transfer between the ER and mitochondria: Intracellular lipid trafficking generally occurs via two modes: vesicular and non-vesicular transport. Because mitochondria are not part of the endomembrane system and therefore cannot receive lipids via vesicular transport, they rely exclusively on non-vesicular mechanisms for lipid acquisition. This process is mediated by lipid transfer proteins (LTPs), which mobilize distinct lipid molecules from source membranes and deliver them across membrane contact sites (MCSs). As a specialized MCS, MAM harbors a diverse array of LTPs and serves as a key conduit for shuttling lipids to mitochondria [19]. Regions of narrow spacing between the ER and mitochondria (10 nm) have been shown to mediate this process [20]. (3) Anchoring key regulators of lipid metabolism: A series of proteins localized to MAM specifically regulate lipid metabolism.

The subsequent sections explore the role of MAM in controlling the metabolism of key lipid categories, such as PLs, sphingolipids, CHOL, FAs, and TGs, as well as in maintaining LD balance. Rather than implying that all lipid abnormalities are intrinsically nephrotoxic, we highlight the specific contexts in which altered lipid metabolism may contribute to renal ELD and injury. LDs are discussed separately because they are dynamic organelles that store neutral lipids and reflect the balance between lipid synthesis, storage, mobilization, and degradation.

### MAM regulation of phospholipid and sphingolipid metabolism

PL metabolism is tightly orchestrated in the MAM, which enriches key biosynthetic enzymes and LTPs to facilitate directed synthesis and non-vesicular transport between the ER and mitochondria [19,21]. The core enzymes in the MAM include phosphatidylserine synthases (PSS1/2), phosphatidylethanolamine N-methyltransferase (PEMT), and ethanolamine phosphotransferase (EPT). Specifically, PSS1/2 drives phosphatidylserine (PS) synthesis, PEMT mediates phosphatidylethanolamine (PE) methylation to form phosphatidylcholine (PC), and EPT contributes to PC biosynthesis, collectively laying the foundation for PL balance [21,22].

LTPs at the MAM ensure targeted PL delivery to the mitochondria, which is essential for mitochondrial functions. Oxysterol-binding protein-related protein 5/8 (ORP5/8) complexes, mitofusin 2 (MFN2), and mitoguardin 2 (MIGA2) are pivotal for PS transport. ORP5/8-mediated PS transfer has been demonstrated *in vitro* in HeLa cells, the lipid transfer activity of MIGA2 has been characterized in cell-free systems by structural analyses and liposome reconstitution, and the MFN2-dependent regulation of MAM PS transport has been shown *in vivo* in liver-specific Mfn2 knockout mice and primary mouse hepatocytes [23–25]. Phosphotyrosine interaction protein 51 (PTPIP51) facilitates the transfer of phosphatidic acid (PA) to the mitochondria, serving as a precursor for cardiolipin (CL), a mitochondria-specific PL critical for membrane stability [26]. Steroidogenic acute regulatory protein-related lipid transfer domain-containing protein 7 (StarD7) mediates PC transport from the ER to mitochondria, as demonstrated *in vitro* in mouse C2C12 myoblasts and human primary skeletal myoblasts [27]. Its potential impact on renal lipid homeostasis via fatty acid oxidation (FAO) regulation remains an extrapolation, as no direct renal evidence is currently available [28].

**Figure 1:**
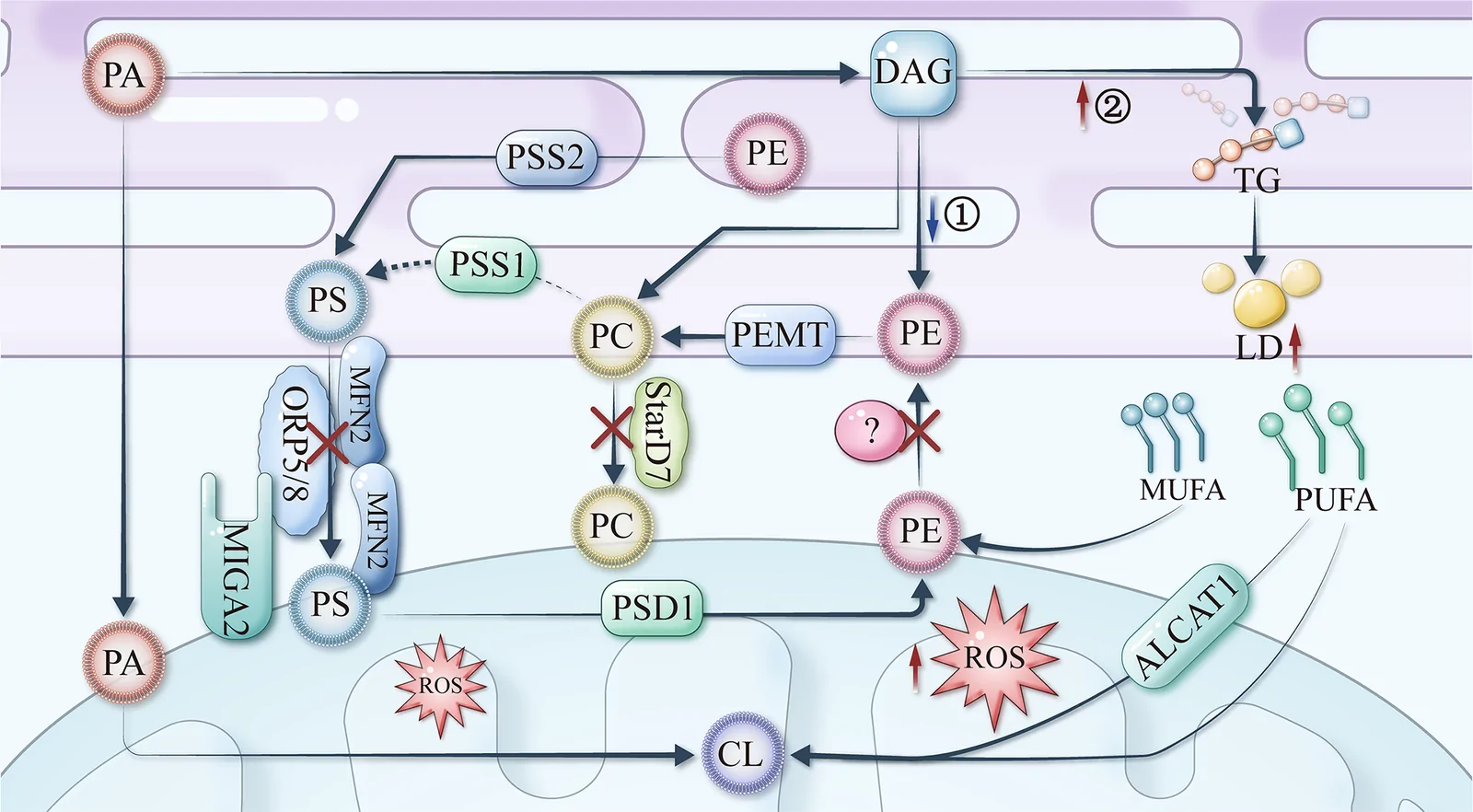
MAM Regulation of PL Metabolism MAM serves as a central hub for PL synthesis, remodeling, and ER-mitochondria PL transport. PS is synthesized in the ER by PSS1/2 and transported to mitochondria via ORP5/8, MFN2, and MIGA2, where it is decarboxylated to PE by PSD1; PE then returns to the ER for methylation to PC by PEMT, and PC is delivered to mitochondria by StarD7. PTPIP51 facilitates PA transport to mitochondria for CL synthesis. ALCAT1-mediated incorporation of PUFA into CL promotes ROS generation and mitochondrial dysfunction. Accumulation of PS may divert lipid metabolism toward neutral lipids, promoting DAG-TG synthesis and LD formation. ALCAT1, acyl-CoA:lysocardiolipin acyltransferase-1; CL, cardiolipin; DAG, diacylglycerol; LD, lipid droplet; MFN2, mitofusin-2; MIGA2, mitoguardin 2; MUFA, monounsaturated fatty acid; ORP5/8, oxysterol-binding protein-related protein 5/8; PA, phosphatidic acid; PC, phosphatidylcholine; PE, phosphatidylethanolamine; PEMT, phosphatidylethanolamine N-methyltransferase; PS, phosphatidylserine; PSD1, phosphatidylserine decarboxylase 1; PSS1/2, phosphatidylserine synthase 1/2; PTPIP51, protein tyrosine phosphatase-interacting protein 51 (also referred to as PTIPIP51); PUFA, polyunsaturated fatty acid; ROS, reactive oxygen species; StarD7, StAR-related lipid transfer domain-containing protein 7; TG, triglyceride.

Taken together, MAM is enriched with both PL-synthesizing and remodeling enzymes and harbors various proteins involved in the transfer of PLs between the ER and mitochondria (Figure 1). The accumulation of PS in the ER feedback inhibits PSS1/2, shifting PL synthesis toward neutral lipids, such as TGs, while altered PL transfer to LDs promotes LD fusion and ELD [21,29]. Acyl-CoA:lysocardiolipin acyltransferase-1 (ALCAT1) is the only PL-remodeling enzyme identified in renal MAM. It exacerbates pathology by incorporating polyunsaturated fatty acids (PUFAs) into CL, resulting in mitochondrial dysfunction, oxidative stress, and podocyte injury in DN. This was demonstrated in high-glucose-treated cultured podocytes, where ALCAT1 silencing reduced oxidized CL and mitochondrial oxidative stress [30]. The pro-lipotoxic role of ALCAT1-mediated CL remodeling was first established in obese mice [31].

PLs are indispensable structural and signaling components of cellular membranes and are not inherently toxic. Their pathological effects on the kidney are more likely to arise from abnormal PL composition, defective inter-organelle trafficking, impaired CL remodeling, or the generation of peroxidation-prone PL species. Similarly, ceramide accumulation within the sphingolipid category is most strongly associated with renal lipotoxicity. In contrast, the renal effects of changes in other sphingolipid species are not fully understood.

Beyond PLs, sphingolipids, particularly ceramides (Cer), emerge as key mediators of renal lipotoxicity, with elevated levels linked to AKI, DN, and fibrosis [32,33]. MAM directly regulates Cer biosynthesis through core enzymes that drive *de novo* synthesis: serine palmitoyltransferase (SPT), ceramide synthase (CerS), and dihydroceramide desaturase (DES1) [34–36]. SPT catalyzes the rate-limiting step, forming a functional complex at the MAM via the mitochondrial subunit SPTLC2 and ER-resident SPTLC1 [34]. Among CerS isoforms, CerS6 is enriched in the MAM and plays a pathogenic role in the kidney. *In vivo*, podocyte-specific overexpression of CerS6 in mice increases mitochondrial Cer levels, triggering mitochondrial DNA release and inflammatory injury, whereas podocyte-specific CerS6 knockout ameliorates glomerular injury in two diabetic mouse models and in adriamycin-induced nephropathy mice [37]. In the renal tubular compartment, Cers6-deficient mice and CERS6-knockdown high-glucose HK-2 cells both show attenuated mitochondrial damage and interstitial fibrosis in diabetes [38]. Responsible for completing Cer synthesis by introducing a double bond into dihydroceramide, it modulates renal pathology through the suppression of the nuclear factor kappa-light-chain-enhancer of activated B cells pathway. Its inhibition reduces inflammation and fibrotic signaling *in vitro* in cultured renal and cardiac cells exposed to uremic toxins [39]. However, corresponding *in vivo* renal validation remains absent.

**Figure 2:**
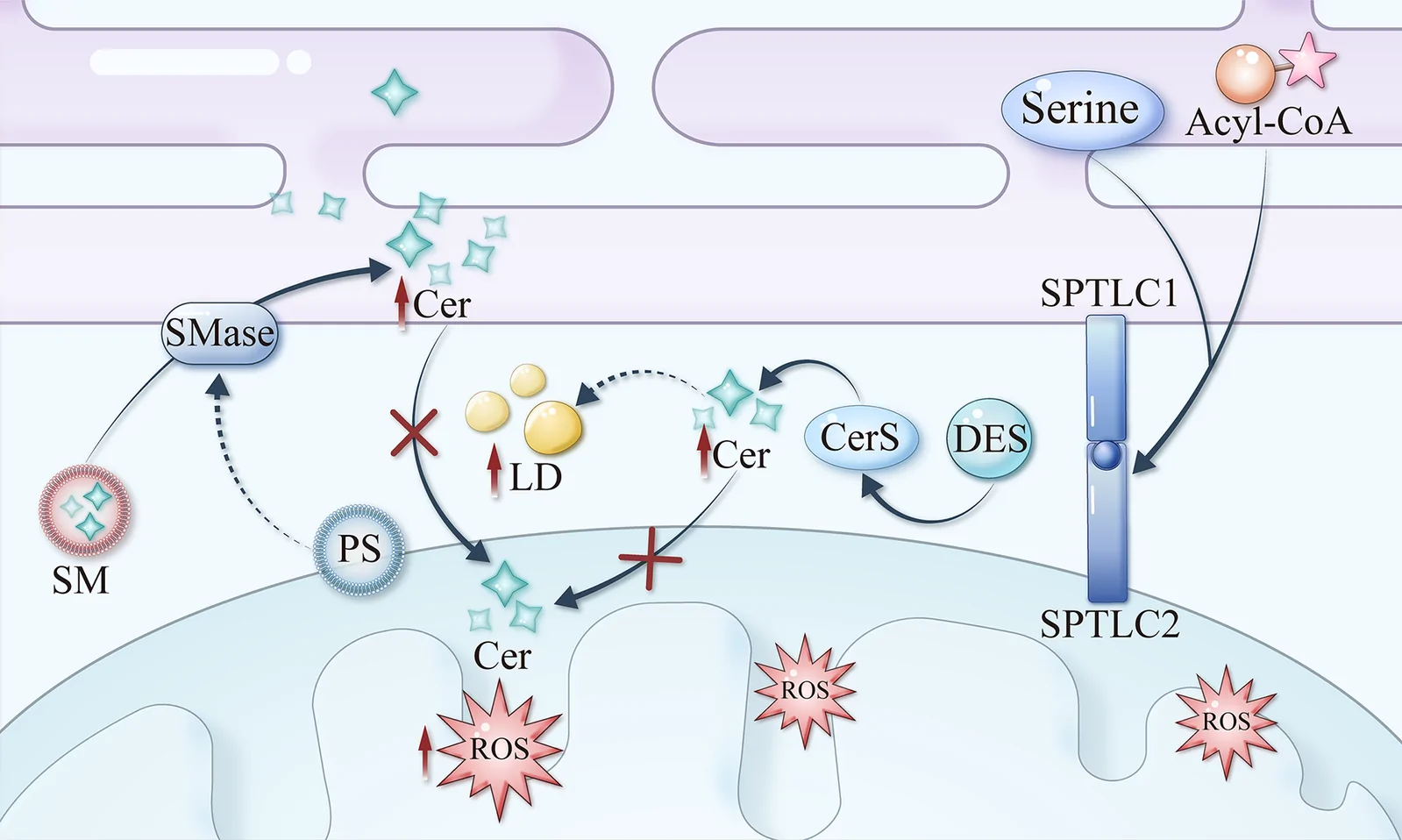
MAM Regulation of Sphingolipid Metabolism MAM is involved in both the *de novo* synthesis of Cer from Serine and Acyl-CoA via SPTLC1/2, CerS, and DES, and the generation of Cer through SM hydrolysis by SMase. Cer accumulation may promote LD formation and contribute to PS perturbation and ROS production in renal cells. Acyl-CoA, acyl coenzyme A; Cer, ceramide; CerS, ceramide synthase; DES, dihydroceramide desaturase (also referred to as DEGS); LD, lipid droplet; PS, phosphatidylserine; ROS, reactive oxygen species; Serine, L-serine; SM, sphingomyelin; SMase, sphingomyelinase; SPTLC1/2, serine palmitoyltransferase long chain base subunit 1/2.

Ceramide production also arises from sphingomyelin (SM) hydrolysis by acid sphingomyelinase (aSMase) and neutral sphingomyelinase (nSMase), with MAM implicated in this process [40]. Although renal MAM localization of these SMases remains unconfirmed, aSMase knockout mice show alleviated glomerular endothelial injury in sepsis-associated AKI [41], and pharmacological aSMase inhibition (α-mangostin) reduces ERS and apoptosis in the kidneys of Goto-Kakizaki diabetic rats [42]. These findings highlight the potential crosstalk between MAM and SM hydrolysis in renal lipotoxicity (Figure 2). Furthermore, MAM dysfunction can perturb sphingolipid metabolism through bidirectional regulation. In neural cells, the pathological accumulation of MAM triggers the activation of nSMase, which subsequently increases Cer levels [43]. In contrast, DES1 deficiency impairs MAM structure and function in mouse brain tissue, likely because sphingolipids maintain the MAM membrane composition [36]. These findings underscore that MAM-mediated sphingolipid dysregulation contributes to Cer accumulation and subsequent renal injury, whether it occurs via *de novo* synthesis or SM hydrolysis. However, direct evidence in kidney-specific contexts remains to be investigated.

### MAM regulation of cholesterol metabolism

CHOL is an essential structural component of cellular membranes; however, excessive intracellular accumulation can exert cytotoxic effects by disrupting membrane fluidity, impairing membrane protein function, and causing other detrimental cellular events [44]. Wang et al. demonstrated *in vitro* using the mouse collecting duct cell line mpkCCD that CHOL up-regulates pro-oxidant enzymes and induces a loss of mitochondrial membrane polarization, leading to increased intracellular reactive oxygen species (ROS) levels, which could be mitigated by statin treatment. They further observed related protective effects *ex vivo* in tubular suspensions of inner medullary collecting duct cells from rat kidneys and *in vivo* in 5/6 nephrectomized (5/6Nx) rats fed a high-fat diet (HFD) [45]. Kong et al. further reported that by integrating human, *in vitro*, and *in vivo* data, CHOL activates the NOD-like receptor family pyrin domain-containing 3 (NLRP3) inflammasome, leading to the release of interleukin-1β, which subsequently down-regulates aquaporin-2 (AQP2) expression and alters renal water and sodium handling [46]. Their work included a retrospective cohort analysis of human kidney biopsy specimens showing a negative correlation between serum CHOL and AQP2 expression as well as estimated glomerular filtration rate, *in vitro* CHOL treatment experiments in renal cells, studies in NLRP3 knockout mice on an HFD, and experiments in 5/6Nx rats on HFD treated with atorvastatin. Similar to these findings, Qiu et al. confirmed in HK-2 cells, the human proximal tubular epithelial cell line, that CHOL induces ERS and apoptosis [47]. Interestingly, while cholesteryl ester (CE) is typically regarded as a benign storage form of CHOL, recent evidence from animal models, particularly in uninephrectomized mice fed a high-fat diet for 13 weeks, and cellular studies of renal lipid deposition suggest that enhanced cholesteryl esterification contributes to renal lipid deposition and represents a key source of lipotoxicity in the kidney, as shown *in vivo* by targeted lipidomics in podocytes of high-fat-diet uninephrectomized mice [48]. Given the critical role of CHOL dysregulation in renal cellular injury, it is essential to examine how MAM contributes to CHOL regulation in this context.

MAM acts as a lipid raft-like domain highly enriched in CHOL, with reported levels 5-7 times higher than those found in mitochondria and microsomes [49]. This unique lipid composition may necessitate localized CHOL biosynthesis to support the formation and maintenance of MAMs. Consistent with this, several key enzymes involved in CHOL metabolism, including 3-hydroxy-3-methylglutaryl-CoA reductase (HMG-CoAR) [18], squalene synthase (SQS), and squalene epoxidase (SQLE), are enriched in MAM [50]. MAM also regulates CHOL synthesis through the SREBP2 signaling axis. SREBP2, a transcriptional regulator of CHOL biosynthesis, promotes the expression of target genes such as HMG-CoAR and low-density lipoprotein receptor (LDLr) [51]. Under basal conditions, SREBPs are retained in the ER membrane by binding to insulin-induced gene 1 (Insig-1). When ER CHOL levels drop, this complex dissociates and translocates to the Golgi, where SREBP2 undergoes proteolytic cleavage, allowing the active nuclear fragment (nSREBP2) to activate the transcription of CHOL-related genes [51,52]. Notably, MAM has been identified as a potential upstream regulator of this pathway through various MAM-associated proteins, such as sigma-1 receptor (Sig-1R) [53]. In hepatocytes with hepatic steatosis and preclinical diabetic kidney models, elevated Sig-1R expression has been observed alongside MAM structural disruption [25,53]. At the cellular mechanistic level, Sig-1R has been shown to participate in the ER-associated degradation pathway in mammalian cell models, promoting the dissociation of Insig-1 from SCAP and thereby activating SREBP signaling [54].

Moreover, proteins such as oxysterol-binding protein-related protein 5 (ORP5) and ERLIN1/2 (endoplasmic reticulum lipid raft-associated protein 1/2) play a role in regulating SREBP activity, with ORP5 enhancing and ERLIN1/2 stabilizing the Insig-1/SCAP/SREBP complex [55,56]. Concurrently, mechanisms associated with the mitochondria-associated MAM indicate that post-translational modifications of enzymes involved in CHOL biosynthesis also influence the regulation of CHOL synthesis. For instance, research indicates that the mitochondrial enzyme aldehyde dehydrogenase 2 (ALDH2) promotes the ubiquitin-mediated degradation of HMG-CoAR under conditions of excess CHOL in hepatocytes via MAM [57].

Although the kidney is not primarily known for producing steroids, research has revealed the movement of CHOL from the ER to mitochondria in kidney cells, suggesting the role of certain lipid transport proteins. Steroidogenic acute regulatory protein (StAR) and other CHOL transport proteins have been demonstrated to mediate CHOL transfer under specific conditions [58]. Renal MAM may possess the capability to perform this transfer, although direct investigations are currently lacking. Notably, StAR expression is down-regulated in the kidneys of streptozotocin-induced diabetic male Wistar rats, indicating the potential impact of renal MAM dysfunction on CHOL transport and steroidogenesis [59].

Additionally, CHOL efflux in the kidneys predominantly relies on transporters, such as ABCA1 (ATP-binding cassette subfamily A member 1) and ABCG1 (ATP-binding cassette subfamily G member 1) [8]. Of these two efflux transporters, ABCG1 has been identified as a MAM-resident protein in mammalian neural tissues via quantitative proteomic analysis, and its down-regulation correlates with intracellular CHOL accumulation in neurodegenerative pathological states [60]. In CKD, dysregulated renal ABCG1 expression is strongly associated with ectopic intrarenal CHOL deposition [8]. Although the functional role of MAM in CHOL efflux remains to be fully elucidated in renal cells, existing evidence suggests that it can serve as a platform for proteins such as ABCG1, thereby regulating CHOL metabolism and its efflux.

**Figure 3:**
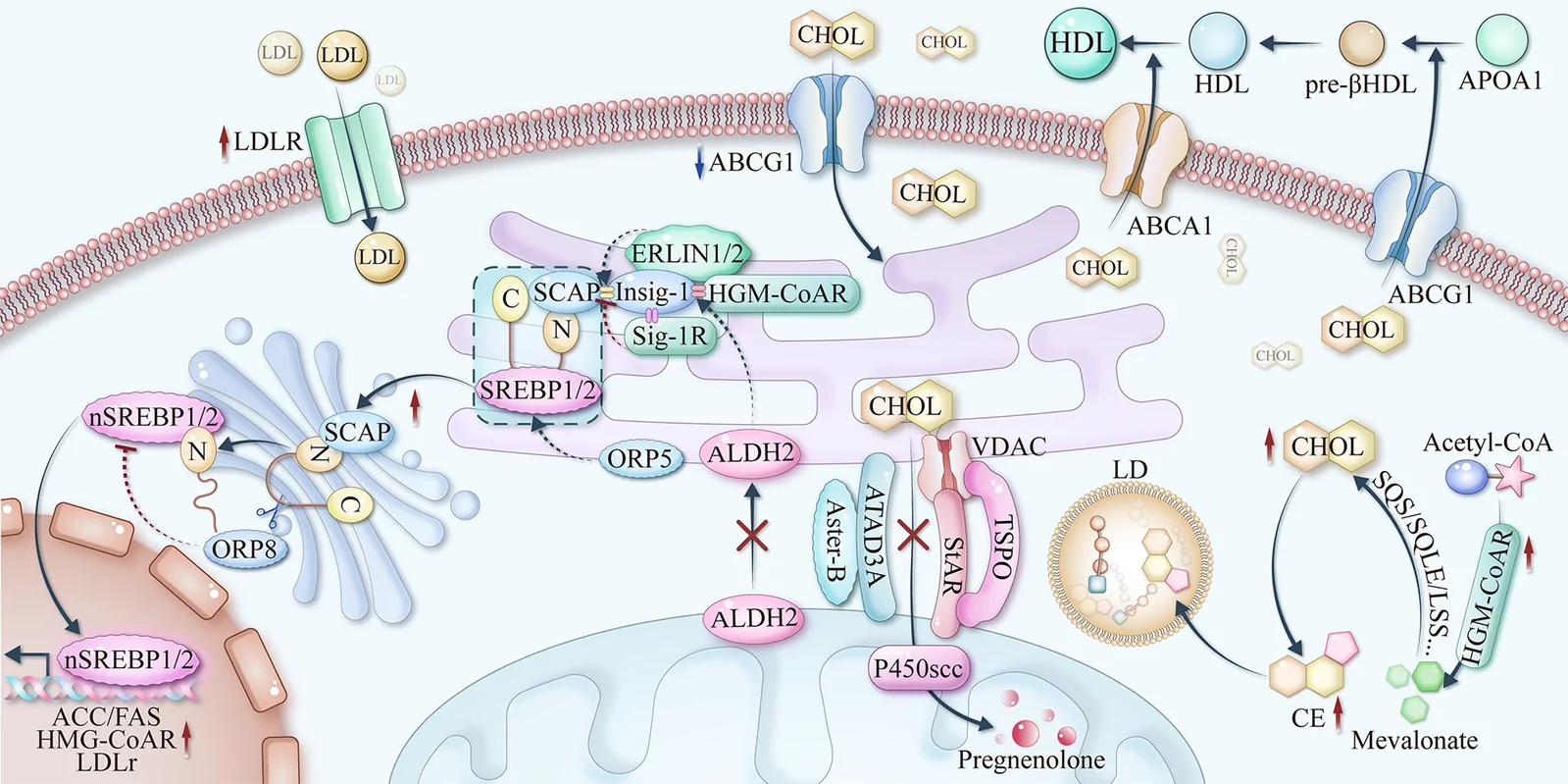
MAM Regulation of CHOL Metabolism MAM is enriched with proteins involved in CHOL synthesis (HMG-CoAR, SQS, and SQLE), SREBP-mediated transcriptional regulation (SREBP1/2-SCAP-INSIG-1, with ERLIN1/2, Sig-1R, and ORP5), LDL (low-density lipoprotein) uptake via LDLr, and CHOL efflux via ABCA1 and ABCG1 to HDL. ALDH2 promotes HMG-CoAR degradation at the post-translational level. StAR mediates CHOL transport from the ER to the mitochondria. MAM dysfunction may lead to abnormal CHOL accumulation and impaired mitochondrial trafficking, thereby contributing to renal lipotoxicity. ABCA1, ATP-binding cassette subfamily A member 1; ABCG1, ATP-binding cassette subfamily G member 1; Acetyl-CoA, acetyl coenzyme A; ALDH2, aldehyde dehydrogenase 2; CE, cholesteryl ester; CHOL, cholesterol; ERLIN1/2, endoplasmic reticulum lipid raft-associated protein 1/2; HDL, high-density lipoprotein; HMG-CoAR, 3-hydroxy-3-methylglutaryl-coenzyme A reductase; INSIG-1, insulin-induced gene 1 protein; LD, lipid droplet; LDL, low-density lipoprotein; LDLr, low-density lipoprotein receptor; Mevalonate, mevalonic acid; nSREBP1/2, nuclear sterol regulatory element-binding protein 1/2; ORP5, oxysterol-binding protein-related protein 5; SCAP, SREBP cleavage-activating protein; Sig-1R, sigma-1 receptor; SQS, squalene synthase (also referred to as SS); SQLE, squalene epoxidase; SREBP1/2, sterol regulatory element-binding protein 1/2; STAR, steroidogenic acute regulatory protein (also referred to as StAR or STARD1).

In conclusion, MAM dysfunction may perturb CHOL biosynthesis, intracellular transport, and efflux, thereby predisposing renal cells to pathological CHOL accumulation and injury (Figure 3). Nevertheless, CHOL is essential for membrane organization and cellular function; thus, renal toxicity is mainly associated with excessive or improperly compartmentalized free CHOL rather than CHOL metabolism *per se*. The kidney-specific contribution of MAM to these processes requires further investigation in the future.

### MAM regulation of fatty acid and triglyceride metabolism

Disrupted free fatty acid (FFA) metabolism and elevated FFA levels are widely observed in kidney diseases [61,62]. Accumulating evidence indicates that FFAs exert potent cytotoxic effects on renal cells. Specifically, excess FFAs can trigger ERS and oxidative stress, leading to mitochondrial dysfunction and additional cellular impairments, ultimately compromising cell viability and even inducing apoptosis [47,63]. To mitigate FFA-induced toxicity, renal cells adopt multiple metabolic strategies to reduce intracellular lipid burden and prevent damage. Among the primary processes are FAO, which mainly occurs within the mitochondria, and TG synthesis, which takes place in the ER [64]. Positioned at the interface between these two organelles, the MAM serves as a key regulatory hub that coordinates and integrates both metabolic routes. To enter either the oxidative or storage pathways, FFAs must first be converted to their active form, acyl-CoA. This reaction is catalyzed by acyl-CoA synthetases, among which acyl-CoA synthetase long-chain family member 4 (ACSL4) is the most well-characterized MAM-associated isoform [65]. Its localization at the MAM suggests that ACSL4 facilitates efficient substrate transfer to adjacent organelles, thereby functioning as a key regulator of activated FA toward specific metabolic pathways. ACSL4 has been experimentally identified in the MAM fractions isolated from the renal cortical tissue of diabetic mice, with complementary validation in HK-2 cells [66], providing direct kidney-specific evidence.

FAO is the dominant energy-generating pathway in renal cells, particularly in tubular epithelial cells [67]. Upon activation, acyl-CoA is first converted to acylcarnitine by carnitine palmitoyltransferase 1 (CPT1) in the OMM. It is then transported across the inner membrane by carnitine-acylcarnitine translocase (CACT). Finally, it is reconverted to acyl-CoA in the mitochondrial matrix by carnitine palmitoyltransferase 2 (CPT2). The resulting acyl-CoA then undergoes sequential β-oxidation catalyzed by acyl-CoA dehydrogenases [28]. Recent studies suggest that the MAM acts as a spatial and functional coordinator of FAO by integrating key enzymatic components. Pang et al. proposed the existence of a carnitine palmitoyltransferase 1A/VDAC (voltage-dependent anion channel)/ACSL protein complex localized at the MAM, which facilitates the import of activated FAs into the mitochondria for β-oxidation [68]. This structural arrangement may enable efficient metabolic channeling between organelles and support optimal FAO under physiological conditions. Under pathological conditions such as hyperglycemia, mitochondrial quality and function are often impaired, resulting in defective FAO [67]. This dysfunction contributes to ELD and triggers a compensatory shift from FAO to glycolysis, known as metabolic reprogramming. Metabolic reprogramming is a hallmark of kidney disease and a potential driver of AKI-to-CKD transition [2,67,69]. Consequently, MAM dysfunction may hinder FAO, resulting in intracellular lipid accumulation and renal injury.

In addition to FAO, TG synthesis is another major metabolic fate of FFAs. Once converted to acyl-CoA, FFAs enter the glycerol-3-phosphate pathway. In this pathway, glycerol-3-phosphate is sequentially acylated by glycerol-3-phosphate acyltransferase (GPAT) and 1-acylglycerol-3-phosphate acyltransferase (AGPAT) to form PA. PA is then dephosphorylated by lipin to generate diacylglycerol (DAG). Diacylglycerol acyltransferase 2 (DGAT2) catalyzes the final step, esterifying DAG with a third acyl-CoA to form TG, and is widely reported to be localized at the MAM [70]. This positions the MAM as a critical site for integrating FA activation with TG synthesis and storage in LDs. In addition to storage in LDs, newly synthesized TG can be assembled into VLDL (very-low-density lipoprotein) particles for secretion in certain cells, offering an alternative route to alleviate intracellular lipid overload [71]. This process has been increasingly linked to the MAM, which serves as a key regulatory site for VLDL biogenesis. Vance et al. reported the enrichment of lipoproteins within the hepatic MAM, proposing a critical role in hepatic lipoprotein synthesis and secretion. In 2021, Anastasia et al. identified a specialized form of MAM, known as the wrappER, characterized by a curved rough ER tightly enveloping mitochondria and involved in hepatic VLDL biogenesis. This structure harbors key VLDL biosynthetic components, including apolipoprotein B (ApoB), microsomal triglyceride transfer protein (MTP), apolipoprotein E (ApoE), and carboxylesterase 1D (Ces1d). Anastasia et al. revealed that structural perturbation of the wrapper-mitochondria contact impairs VLDL biogenesis, resulting in the accumulation of FFAs, TG, and LDs. In the same year, Ilacqua et al. reported that peroxisomes are also partially present within the wrapper-mitochondrial contact structure. Upon disruption of VLDL biogenesis, these peroxisomes, together with mitochondria, coordinately enhance FAO to mitigate excess intracellular FAs [71]. However, this regulatory function appears to be limited to hepatocytes and intestinal epithelial cells and is likely absent in other cell types, such as neurons [72]. Renal cells do not possess an active VLDL secretory pathway, suggesting that MAM-regulated lipoprotein metabolism may be largely absent in the kidneys.

**Figure 4:**
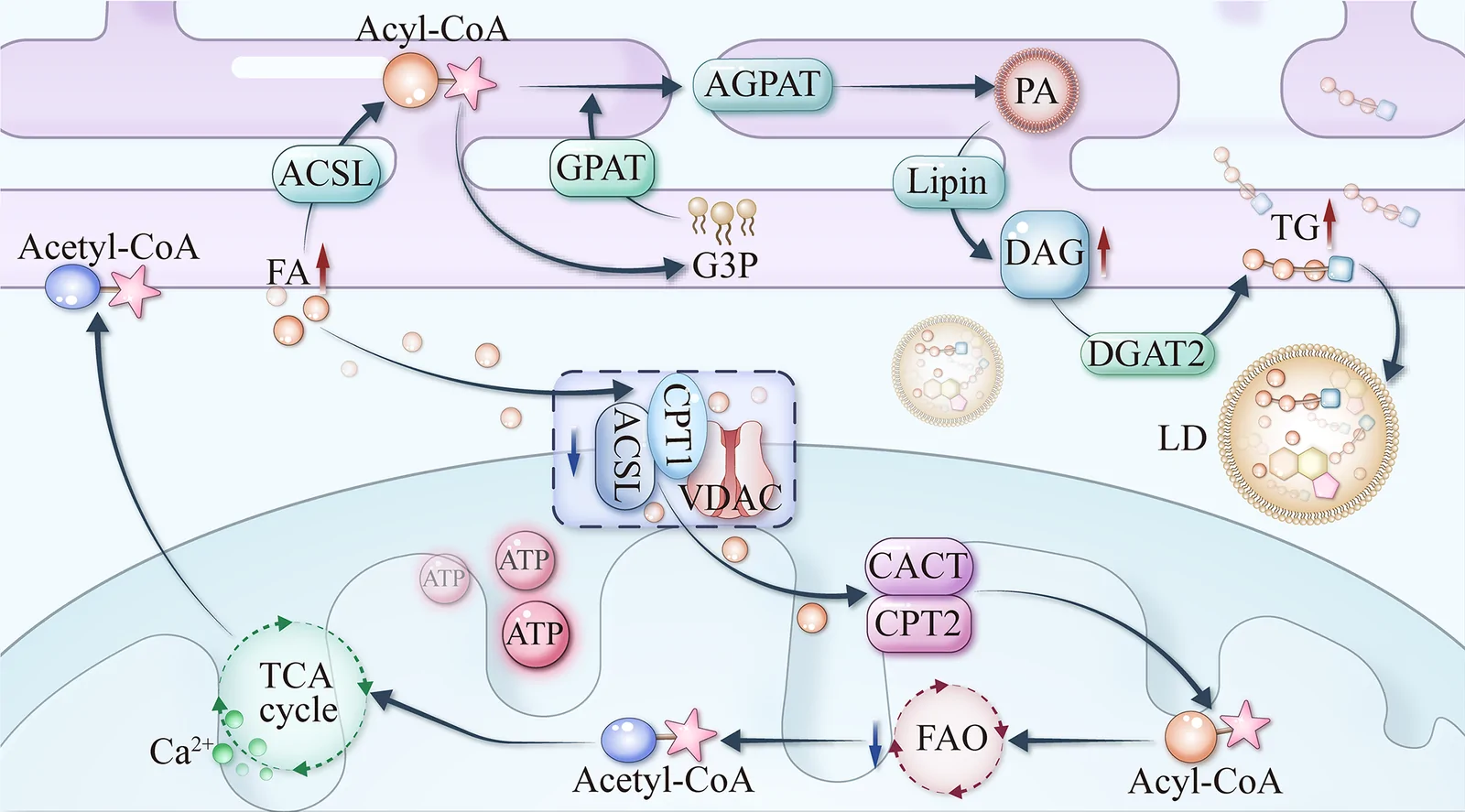
MAM Regulation of FA and TG Metabolism MAM coordinates the two major metabolic fates of FAs: mitochondrial FAO and TG synthesis for LD storage. ACSL (acyl-CoA synthetase long-chain family member) activates FAs in the MAM, and the CPT1/VDAC/ACSL complex facilitates FA entry into the mitochondria, where CACT and CPT2 mediate β-oxidation. TG synthesis proceeds from G3P (glycerol-3-phosphate) through the sequential actions of GPAT, AGPAT, Lipin, and DGAT2. MAM dysfunction may impair these processes, promoting the accumulation of FAs and TGs and contributing to renal injury. Acetyl-CoA, acetyl coenzyme A; ACSL, acyl-CoA synthetase long-chain family member; AGPAT, 1-acylglycerol-3-phosphate O-acyltransferase; ATP, adenosine triphosphate; CACT, carnitine-acylcarnitine translocase; Ca^2+^, calcium ion; CPT1, carnitine palmitoyltransferase 1; CPT2, carnitine palmitoyltransferase 2; DAG, diacylglycerol; DGAT2, diacylglycerol acyltransferase 2; FA, fatty acid; FAO, fatty acid oxidation; G3P, glycerol-3-phosphate; GPAT, glycerol-3-phosphate acyltransferase; LD, lipid droplet; Lipin, phosphatidic acid phosphatase (PAP); PA, phosphatidic acid; TCA cycle, tricarboxylic acid cycle; TG, triglyceride; VDAC, voltage-dependent anion channel.

Collectively, MAM orchestrates multiple aspects of FA handling, including oxidation and storage via TG synthesis (Figure 4). In secretory cells such as hepatocytes, it also participates in lipid export through VLDL assembly. Therefore, in renal cells, MAM dysfunction may impair FAO and alter TG synthesis and storage, contributing to an imbalance between FA utilization and neutral-lipid sequestration. Although excess FFAs are well-recognized mediators of lipotoxicity, TG synthesis and storage can initially serve as protective buffering mechanisms by limiting the availability of toxic FA species. Therefore, TG accumulation is not necessarily injurious; it may become maladaptive when storage capacity is exceeded, lipid-droplet turnover is impaired, or toxic lipid intermediates and oxidative stress persist.

### MAM regulation of lipid droplet metabolism

LDs are dynamic organelles rather than distinct lipid classes and consist of a neutral lipid core surrounded by a PL monolayer. They serve as protective buffers that sequester excess FAs and sterols and provide energy. Accordingly, LD formation is not inherently pathogenic and may initially protect renal cells from lipotoxic free lipid exposure. However, persistent or excessive LD accumulation, particularly when coupled with defective lipolysis, lipophagy, FA oxidation, or mitochondrial function, may reflect maladaptive lipid handling and contribute to renal injury [73]. In the kidney, MAM dysfunction is associated with altered lipid metabolism and increased LD accumulation, which correlates with structural and functional impairments [74]. As LDs establish functional contacts with the ER and mitochondria, both of which are essential for LD biogenesis and turnover, MAM may be important for maintaining their homeostasis. Notably, Najt et al. showed that MAM has structural heterogeneity in hepatic models, with its subdomain adjacent to LDs being compositionally distinct and modulating lipid metabolism via selective protein enrichment [75]. This section discusses the contribution of MAM to LD metabolism, focusing on LD biogenesis and degradation.

MAM regulates LD biogenesis starting in the ER, where neutral lipids such as TG and CEs are synthesized and accumulate. Once their concentration exceeds a threshold, phase separation leads to the formation of lipid lenses, which bud off into the cytoplasm. Seipin is essential for LD biogenesis, determines the sites of neutral lipid lens formation, and interacts with lipogenic enzymes such as GPAT and AGPAT. At the ER-LD contact sites, Seipin facilitates the transfer of proteins and lipids from the ER to LDs during growth, while also maintaining LD homeostasis by preventing excessive fusion [73,76]. Evidence that Seipin deficiency causes congenital generalized lipodystrophy (CGL) with ELD and renal involvement comes from both rodent models and human patients with CGL [77]; Seipin localization at the MAM and its regulation of Ca^2+^ homeostasis were initially characterized *in vitro* in adipocytes [78]. Chi et al. confirmed that Seipin recruitment to the MAM relies on its interaction with the IP3R (inositol 1,4,5-trisphosphate receptor)/GRP75 (glucose-regulated protein 75)/VDAC complex, which is essential for LD synthesis. Guyard et al. highlighted that ORP5 and oxysterol-binding protein-related protein 8 (ORP8) are crucial for Seipin recruitment at the MAM-LD interface and directly regulate LD biogenesis. Knockdown of either protein delays LD formation and increases enlarged LDs under lipid overload conditions. The regulator, MIGA2, promotes inter-organelle coupling in adipocytes, thereby enhancing TG synthesis efficiency [79]. Although the mechanisms of MAM-regulated LD biogenesis in the kidney have yet to be reported, a significant correlation between MAM disruption and elevated adipose differentiation-related protein/PLIN2 expression has been demonstrated in human renal biopsy samples from patients with DN [17], indicating the potential implications of MAM dysfunction in renal LD biogenesis.

Impaired LD degradation has been implicated in renal ELD [80]. LD degradation occurs through classical lipolysis and lipophagy, with lipolysis serving as a prerequisite for lipophagy by breaking down large LDs into smaller LDs. In classical lipolysis, TG in LDs is hydrolyzed by enzymes such as ATGL (adipose triglyceride lipase) and hormone-sensitive lipase [81]. LD degradation disruption, as observed in ATGL-deficient mice, causes structural and functional renal injury and lipotoxicity [82]. Han et al. reported that suppression of lipophagy in renal tubular cells disrupts the adiponectin receptor 1/AMP-activated protein kinase (AMPK) signaling pathway, exacerbating ELD in DN [83]. However, studies on the regulation of MAM during LD degradation in the kidney are scarce.

**Figure 5:**
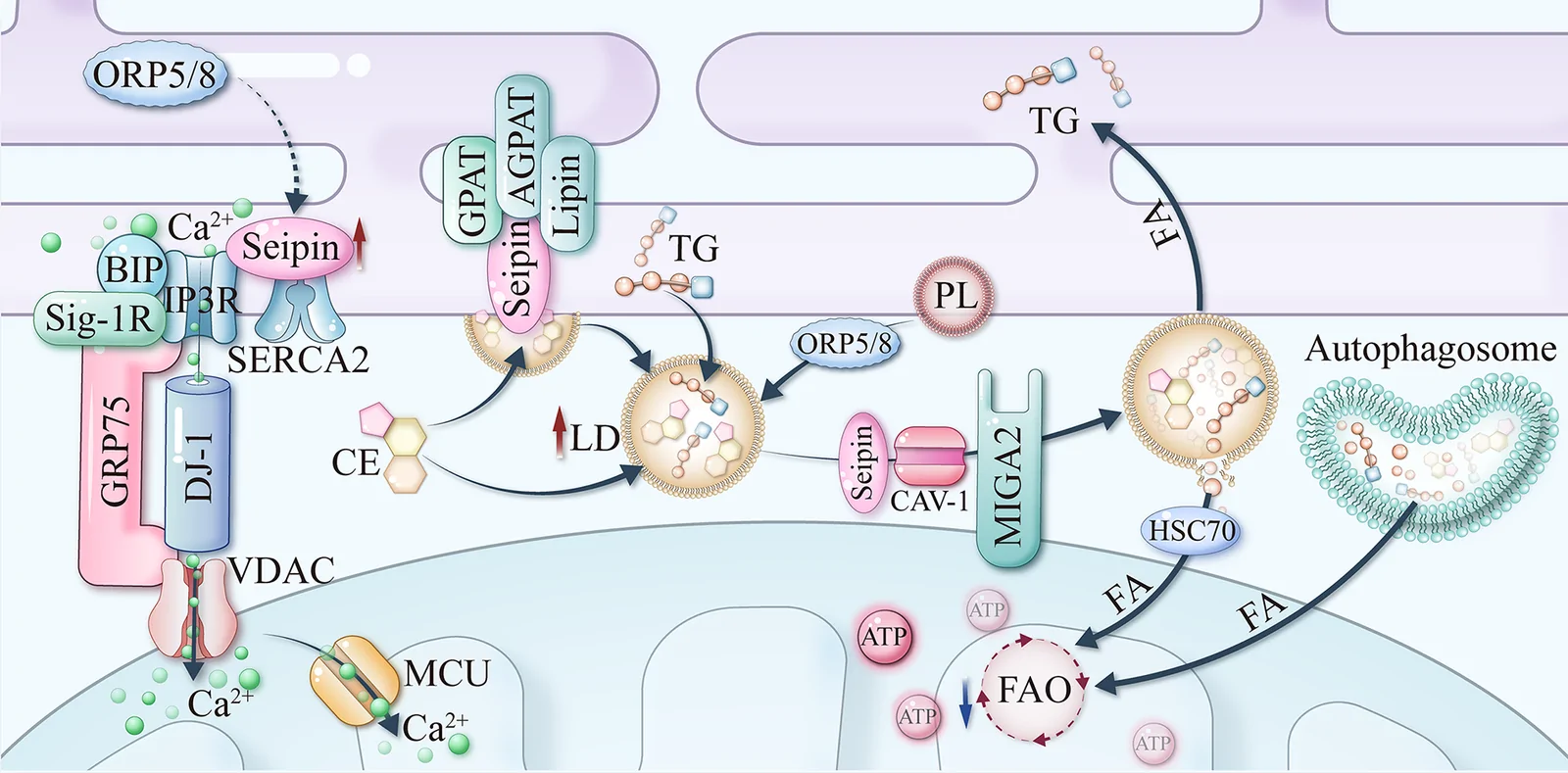
MAM Regulation of LD Metabolism MAM facilitates the spatial and functional integration of mitochondria, the ER, and LDs and coordinates the localization of proteins essential for LD biogenesis. Seipin is recruited to MAM in an IP3R/GRP75/VDAC-dependent manner and interacts with GPAT, AGPAT, and Lipin; ORP5/8 are critical for Seipin enrichment at MAM-LD contacts. MIGA2 promotes organelle coupling and TG synthesis. MAM also provides a platform for lipophagy, whereby LDs are sequestered within autophagosomes and the released FAs enter mitochondrial β-oxidation. MAM dysfunction may lead to increased LD formation and impaired lipophagy, resulting in excessive LD accumulation, while the reduction in FAs entering the oxidative pathway further contributes to renal cell injury. AGPAT, 1-acylglycerol-3-phosphate O-acyltransferase; ATP, adenosine triphosphate; BIP, binding immunoglobulin protein (also referred to as GRP78); CAV-1, caveolin-1; Ca^2+^, calcium ion; CE, cholesteryl ester; DJ-1, Parkinsonism-associated deglycase DJ-1 (also referred to as PARK7); FA, fatty acid; FAO, fatty acid oxidation; GPAT, glycerol-3-phosphate acyltransferase; GRP75, glucose-regulated protein 75 (also referred to as HSPA9); HSC70, heat shock cognate protein 70; IP3R, inositol 1,4,5-trisphosphate receptor; LC3-II, microtubule-associated protein 1A/1B-light chain 3-II; LD, lipid droplet; MCU, mitochondrial calcium uniporter; MIGA2, mitoguardin 2; ORP5/8, oxysterol-binding protein-related protein 5/8; PL, phospholipid; Seipin, BSCL2 (Berardinelli-Seip congenital lipodystrophy type 2 protein); SERCA2, sarco/endoplasmic reticulum calcium ATPase 2; Sig-1R, sigma-1 receptor; TG, triglyceride; VDAC, voltage-dependent anion channel.

MAM provides a structural basis for autophagosome formation and is enriched with proteins such as autophagy-related protein 14 and Beclin1. Additionally, energy stress recruits AMPK to MAM, activating autophagy, indicating the role of MAM in energy sensing [84]. Some MAM proteins participate in lipophagy, with ORP8 known to mediate LD recognition under AMPK activation [85] (Figure 5). LD degradation might be hindered due to impaired autophagosome formation resulting from MAM dysfunction. Studies also propose that MAM-LD contact sites facilitate the transfer of lipolysis-derived FAs into mitochondria for β-oxidation [86]. However, contradictory findings by Najt et al. suggest that mitochondria near LDs support lipogenesis rather than β-oxidation [75]. These insights highlight the complexity of MAM-LD interactions in managing FAs from LD degradation; however, the renal implications of these mechanisms remain poorly understood.

The MAM proteins directly involved in lipid metabolism are summarized in Table 1. In addition to its direct regulatory roles in lipid metabolism, which contribute to renal ELD and injury, MAM may promote ELD and injury by modulating other cellular processes. These include calcium signaling, insulin signaling, inflammation, autophagy, ERS, and mitochondrial dynamics. As these pathways have been extensively reviewed elsewhere [8,87–89], they will not be discussed further in the present review.

**Table 1:** MAM proteins directly involved in lipid metabolism and their functions

Lipid metabolism	MAM proteins	Abbreviation	Function
PL metabolism	Phosphatidylserine synthase	PSS	Catalyze the synthesis of PS [18]
	Phosphatidylethanolamine N-methyltransferase	PEMT	Catalyze PE methylation to generate PC [18]
	Ethanolamine phosphotransferase	EPT	Catalyze the synthesis of PC from cytidine diphosphate-choline [18]
	Oxysterol-binding protein-related proteins 5/8	ORP5/8	Transfer PS from the ER to mitochondria [23]
	Mitofusin 2	MFN2	Transfer PS from the ER to mitochondria [25]
	Mitoguardin 2A2	MIGA2	Transfer PS from the ER to mitochondria [24]
	Protein tyrosine phosphatase interacting protein 51	PTPIP51	Transfer PA from the ER to mitochondria [26]
	Steroidogenic acute regulatory protein-related lipid transfer domain containing protein 7	StarD7	Transfer PC from the ER to mitochondria [27]
	Acyl-coenzyme A:lyso-cardiolipin acyltransferase-1	ALCAT1	Remodel CL [31]
Sphingolipid metabolism	Serine palmitoyltransferase	SPT	Catalyze the formation of 3-ketodihydrosphinganine from serine and palmitoyl-CoA [34]
	(Dihydro) ceramide synthase	CerS	Catalyze the synthesis of (dihydro) ceramide [35]
	Dihydroceramide desaturase	DES (DEGS)	Catalyze the synthesis of ceramide [36]
CHOL metabolism	Steroidogenic acute regulatory protein 1	StAR (STARD1)	Transfer CHOL from the ER to mitochondria [58]
	3-hydroxy-3-methylglutaryl-CoA (HMG-CoA) reductase	HMG-CoAR	Serve as key enzymes for CHOL synthesis [18]
	Oxysterol-binding protein-related proteins 5	ORP5	Promote SREBP2 expression [90]
	Oxysterol-binding protein-related proteins 8	ORP8	Suppress nSREBP2 expression [90]
	Sigma-1R	Sig-1R	Promote SCAP/SREBPs translocation [54]
	ER lipid raft-associated proteins 1 and 2	ERLIN1/2	Stabilize the Insig-1/SCAP/SREBPs complex [55]
	ATP-binding cassette subfamily G member 1	ABCG1	Promote CHOL efflux [60]
FA and TG metabolism	Long-chain Acyl-CoA synthetase 4	ACSL4	Catalyze long-chain FA activation [65]
	Voltage-dependent anion channel	VDAC	OMM channel for acyl-carnitine and calcium transport [68]
	Carnitine palmitoyltransferase 1	CPT1	Catalyst for acyl-carnitine formation in FAO [68]
	Diacylglycerol acyltransferase 2	DGAT2	Catalyze the synthesis of TG from DAG [70]
	Microsomal triglyceride transfer protein	MTP	Load TG and other lipids onto ApoB [10,72]
	Apolipoprotein B	ApoB	Constitute VLDL [10,72]
	Apolipoprotein E	ApoE	Constitute VLDL [10,72]
	Carboxylesterase 1d	Ces1d	Regulate VLDL lipid balance and prevent compositional abnormalities [72]
LD metabolism	Seipin	Seipin	Regulate LD biogenesis [76,91]
	Oxysterol-binding protein-related proteins 5	ORP5	Regulate LD biogenesis and recruit Seipin to MAM [90]
	Oxysterol-binding protein-related proteins 8	ORP8	Regulate LD biogenesis [90] and mediate lipophagy [85]
	Mitoguardin 2	MIGA2	Facilitate LD-ER-mitochondria contact to promote TG synthesis [79]

## MAM dysfunction couples renal ELD with oxidative stress

ELD within renal cells is inherently prone to inducing oxidative stress, a central event in lipid-induced nephrotoxicity. This intimate association stems from the susceptibility of accumulated unsaturated lipids, such as polyunsaturated FA-containing PLs, CHOL, and CEs, to ROS attack under sustained oxidative conditions. Such attacks yield cytotoxic byproducts such as 4-hydroxynonenal and malondialdehyde (MDA) through the process of lipid peroxidation, a major pathological branch of lipid metabolism [92,93]. Crucially, lipid peroxidation is not merely a consequence but an amplifier of injury, driving renal pathology by triggering regulated cell death pathways such as ferroptosis, apoptosis, and pyroptosis, thus presenting a promising therapeutic target [94,95]. Although a direct causal chain from deposition to peroxidation is still being delineated, strong correlative evidence exists. For instance, elevated 4-HNE (4-hydroxynonenal) adduct levels (a marker of lipid peroxidation) alongside increased renal lipid accumulation were observed *in vivo* in diabetic mouse kidneys and validated *in vitro* in cultured renal tubular epithelial cells [67]. Impaired FA oxidation, leading to FFA accumulation, which subsequently promotes lipid peroxidation and tubular ferroptosis, was demonstrated *in vivo* in a cisplatin-induced AKI mouse model [96].

The MAM serves as a pivotal cellular hub that couples these two processes together. First, as the central coordinator of lipid metabolism, MAM dysfunction directly disrupts lipid homeostasis. It promotes the biosynthesis of lipids such as TGs while impairing FA oxidation, leading to the intracellular accumulation of oxidizable substrates, notably polyunsaturated FAs [92]. The MAM-localized enzyme ACSL4 plays a specific role in preferentially activating long-chain PUFAs and incorporating them into PLs, such as PE. This creates peroxidation-susceptible lipid species and positions ACSL4 as a rate-limiting factor in the initiation of lipid peroxidation [65,95,97]. Consequently, ACSL4 deficiency is renoprotective, attenuating lipid peroxidation and ferroptosis in kidney disease models [98,99]. In essence, MAM dysfunction expands the cellular reservoir of lipids that are primed for peroxidation.

Second, as a critical nexus for oxidative stress generation and signaling, MAM dysfunction is intrinsically linked to oxidative damage. Although its causal relationship with ERS in the kidney requires further clarification [100,101], MAM dysfunction is more definitively established as a contributor to mitochondrial oxidative stress [102,103]. This link is logical given the mitochondrion's reliance on MAM for the transfer of essential metabolites, such as lipids and calcium ions. The ER and mitochondria are primary intracellular ROS sources, and the MAM, situated at their interface, harbors a multitude of proteins that regulate redox balance. These proteins can be categorized as follows: (1) calcium-regulatory proteins such as the IP3R/GRP75/VDAC complex and SERCA pumps, where disrupted Ca^2+^ signaling directly induces oxidative stress [104,105]; (2) ER chaperone proteins including BiP, calnexin, calreticulin, endoplasmic reticulum protein 44, and Sig-1R, which are involved in Ca^2+^ signaling and stress response at the MAM [106–111]; (3) ER redox enzymes such as the ROS-generating enzymes ERO1α (endoplasmic reticulum oxidoreductin 1α) and NOX4 (nicotinamide adenine dinucleotide phosphate oxidase 4), alongside ROS-scavenging proteins GPX8 (glutathione peroxidase 8) and thioredoxin-related transmembrane protein 2, which interact with calcium pathways [112–115]; (4) ER stress response proteins, notably PERK (PKR-like ER kinase) and inositol-requiring enzyme 1α, key sensors of the unfolded protein response [112,116]; (5) other tethering and regulatory proteins such as MFN2, PACS2, and selenoprotein N, which are all implicated in oxidative stress regulation and confirmed at the renal MAM [66,116–119].

Importantly, the coupling facilitated by MAM is bidirectional. In addition to lipids and calcium, ROS traverse the MAM interface to enable inter-organelle redox communication. While ER stress-mitochondrial dysfunction is well documented [14,120–122], emerging evidence has revealed retrograde signaling from mitochondria to the ER via MAM. Mitochondria can transmit ROS signals back to the MAM, leading to the oxidation of proteins such as IP3R and influencing local calcium dynamics [123]. Similarly, mitochondrial lipid radicals can be transferred to the ER through MAM-localized channels, illustrating a mechanism for mitigating local oxidative damage [124]. This bidirectional crosstalk underscores the role of MAM as an active redox signaling hub rather than merely a passive structural link.

**Figure 6:**
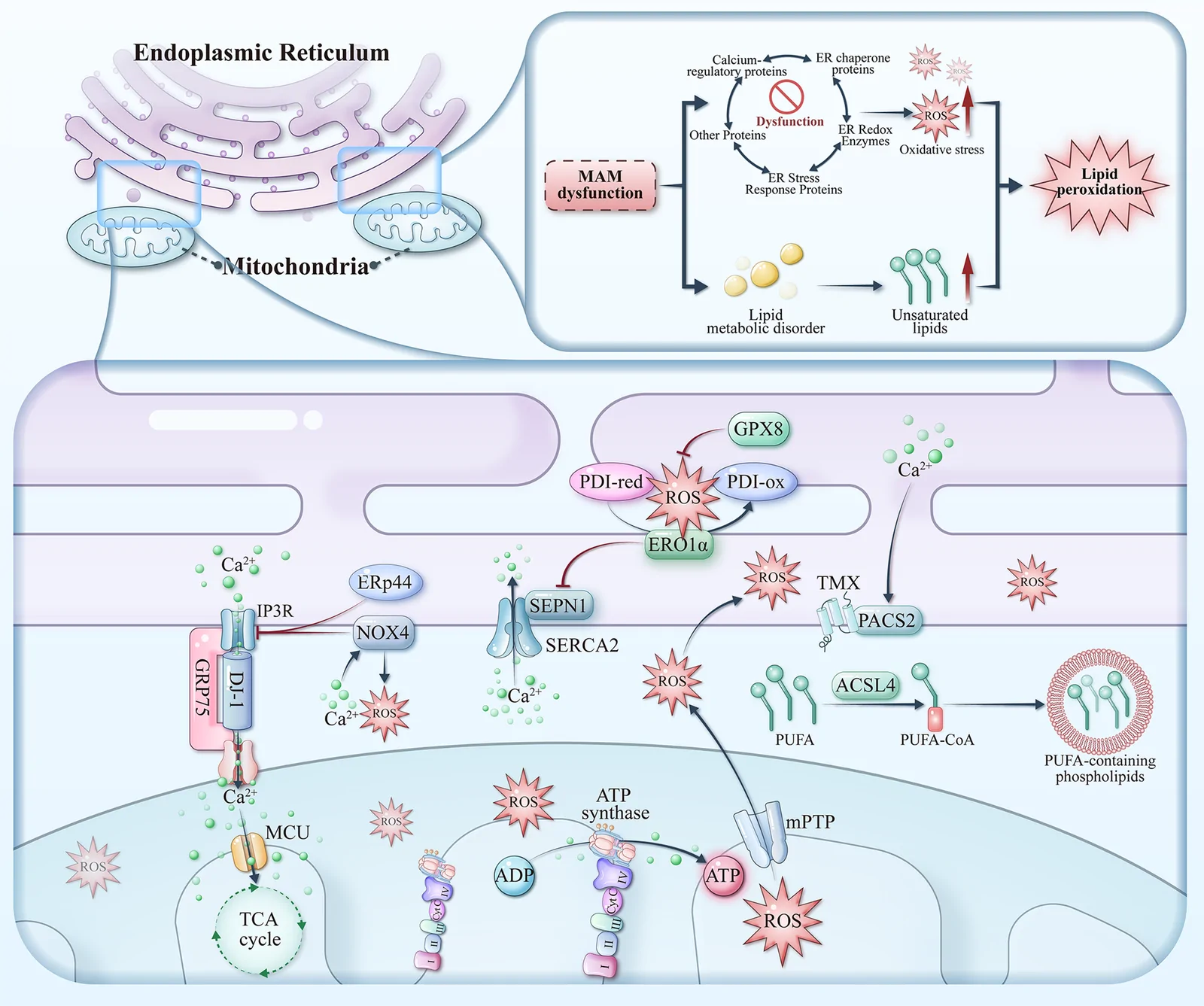
MAM Regulation of Lipid Peroxidation MAM is enriched with oxidative stress-associated proteins, including (1) calcium-regulatory proteins: SERCA, IP3R, GRP75, and VDAC; (2) ER redox enzymes: ERO1α, NOX4, GPX8, and TMX (thioredoxin-related transmembrane protein); and (3) other proteins: MFN2, PACS2, and SEPN1. MAM dysfunction is frequently accompanied by up-regulated oxidative stress and intracellular lipid imbalance, leading to excessive accumulation of PUFAs. ACSL4 at the MAM preferentially activates PUFAs for incorporation into PLs, generating peroxidation-prone substrates that yield cytotoxic products, including 4-HNE and MDA, ultimately promoting apoptosis. Bidirectional ROS crosstalk between the ER and mitochondria, along with mitochondrion-to-ER trafficking of lipid radicals, further amplifies lipid peroxidation. 4-HNE, 4-hydroxynonenal; ACSL4, acyl-CoA synthetase long-chain family member 4; Ca^2+^, calcium ion; ERO1α, endoplasmic reticulum oxidoreductin-1 alpha; GPX8, glutathione peroxidase 8; GRP75, glucose-regulated protein 75 (also referred to as HSPA9); IP3R, inositol 1,4,5-trisphosphate receptor; MDA, malondialdehyde; MFN2, mitofusin-2; NOX4, nicotinamide adenine dinucleotide phosphate oxidase 4; PACS2, phosphofurin acidic cluster sorting protein 2; PL, phospholipid; PUFA, polyunsaturated fatty acid; PUFA-CoA, PUFA-coenzyme A conjugate; ROS, reactive oxygen species; SEPN1, selenoprotein N; SERCA2, sarco/endoplasmic reticulum Ca^2+^-ATPase 2; TMX, thioredoxin-related transmembrane protein.

In summary, MAM functions as a critical coupling unit in renal lipotoxicity. This concurrently drives the accumulation of oxidizable lipid substrates and potentiates the oxidative environment necessary for peroxidation (Figure 6). This creates a self-perpetuating cycle. MAM dysfunction promotes lipid deposition and induces oxidative stress. The resulting lipid peroxidation damages cellular components, including MAM. This further exacerbates metabolic and redox dysfunction, deepening the injury [67,92,96,102,103]. Therefore, MAM is at the confluence of lipid dysmetabolism and oxidative damage, making its integrity a central determinant of cellular fate. Therapeutic strategies aimed at disrupting this deleterious coupling by targeting MAM stability and function hold significant promise for alleviating lipid-induced renal injuries.

## Therapeutic strategies targeting MAM in lipid-induced kidney disease

As previously discussed, multiple kidney diseases, including AKI, DN, CKD, NS, and RCC, are characterized by lipotoxicity-induced injuries. Given the involvement of MAM dysfunction in both ELD and lipid peroxidation-associated renal damage, MAM has attracted attention as a promising therapeutic target for renal protection in patients with diabetes. In this section, we discuss treatment strategies for lipid-induced renal injury from two perspectives: candidate agents directly targeting MAM and MAM-related mechanisms of renoprotective drugs.

### Potential agents targeting MAM

ER-OMM tethering proteins are essential for preserving MAM integrity, and their expression levels critically determine MAM stability and its function. MFN2 serves as a key regulator of this process [104]. Evidence indicates that MFN2 can restore MAM integrity in renal tubular epithelial cells exposed to cisplatin while concurrently interacting with PERK and IRE1 (inositol-requiring enzyme 1) to suppress ERS and promote cell survival [100]. In high-glucose-induced diabetic models, MFN2 overexpression mitigates MAM disruption in podocytes and inhibits the activation of the PERK signaling pathway [125]. MFN2 has also been reported to inhibit lipogenesis and LD accumulation in clear cell renal carcinoma [126]. Several factors have been implicated in the regulation of MAM integrity by MFN2. Disulfide-bond A oxidoreductase-like protein (DsbA-L) is an MAM-associated protein that ameliorates lipid-induced renal injury [17,127]. Its overexpression enhances MFN2 expression and reverses MAM disruption under hyperglycemic conditions in db/db diabetic mice and in tubular cells treated with high glucose levels [128]. In addition, the classical ERS inhibitor 4-PBA (4-phenylbutyric acid) induces MFN2 expression in the kidneys of rats exposed to 3-chloro-1,2-propanediol (3-MCPD), thus restoring MAM integrity and attenuating ERS and mitochondrial dysfunction [101].

In addition to MFN2, PACS-2 (phosphofurin acidic cluster sorting protein 2) is an important tethering protein that contributes to MAM stability and renal protection. It alleviates lipid-induced renal injury and restores MAM integrity in both diabetic mouse kidneys and HK-2 cells [103,129,87]. Recent evidence indicates that mitogen-activated protein kinase 1 (MAPK1) contributes to MAM disruption in DN by down-regulating PACS-2 expression, thereby impairing mitochondrial function and promoting renal injury [66].

The IP3R/GRP75/VDAC complex, a key calcium transfer module at the MAM, is crucial for ER-mitochondria coupling in the kidneys [104]. Reticulon-1A (RTN1A), an ER protein enriched in the MAM, exacerbates MAM contact formation and reduces the ER-mitochondria distance in renal tubular cells from diabetic mice exposed to high glucose levels, as demonstrated both *in vivo* in diabetic mice and *in vitro* in tubular cells cultured under high-glucose conditions [14]. Furthermore, STING (stimulator of interferon genes) functions noncanonically by interacting with VDAC2 (voltage-dependent anion channel 2) to disrupt the GRP75-VDAC2 complex, thereby promoting RCC progression, as demonstrated in RCC cell lines and xenograft models. Pharmacological inhibition using STING antagonists has emerged as a promising therapeutic approach for treating RCC [88]. Taurine (TAU), a semi-essential amino acid, down-regulates GRP75 expression in NS models, reversing abnormal MAM accumulation in renal tubules and alleviating ERS [89].

Similarly, the transient receptor potential ankyrin 1 (TRPA1) inhibitor HC-030031 alleviates cisplatin-induced pathological MAM enrichment in AKI models, as validated *in vivo* in cisplatin-AKI mice, with complementary *in vitro* confirmation [120]. Transient receptor potential vanilloid 1 (TRPV1), another member of the transient receptor potential (TRP) superfamily, exerts renoprotective effects through opposing mechanisms. Capsaicin, a TRPV1 agonist, activates the AMPK pathway via transient Ca^2+^ influx, restoring mitochondrial Ca^2+^ homeostasis and glomerular integrity [130]. Chen et al. recently added Canopy fibroblast growth factor signaling regulator 2 (CNPY2) to the list of MAM modulators involved in renal injury. Overexpression of CNPY2 aggravates MAM disruption under diabetic conditions, whereas its knockdown alleviates these changes and decreases ferroptosis [131].

### Existing renoprotective drugs

Understanding how existing renoprotective drugs confer effects at the molecular and organelle levels is crucial. This helps elucidate disease mechanisms, optimize clinical strategies, and uncover new targets for drug repurposing. Although no currently approved renoprotective drugs have been shown to act directly on MAM, emerging evidence indicates that several drugs may influence the expression or activity of MAM-regulatory proteins in the kidney, as summarized below.

Dioscin, a naturally occurring steroidal saponin with pleiotropic biological activities, has demonstrated renoprotective effects in DN models. Mechanistically, it suppresses ERS by inhibiting the PERK and IRE1 pathways, reduces lipid peroxidation, and activates AMPK-mediated autophagy. Notably, Dioscin also up-regulates MFN2 and down-regulates dynamin-related protein 1 expression, thereby limiting mitochondrial fission. This may contribute to the restoration of MAM integrity and mitochondrial function under diabetic conditions [132].

Statins, which act by inhibiting HMG-CoAR, are primarily prescribed for hyperlipidemia and are widely used to reduce cardiovascular risk in patients with CKD [133]. In an early study examining the long-term renal effects of statin therapy, Corsetti et al. reported that rosuvastatin significantly up-regulated GRP75 expression in both the tubular and glomerular compartments of the kidney. It also exerts protective effects by alleviating ERS and reducing apoptosis [134]. It can be speculated that statins may exert renal benefits partly via GRP75-related MAM regulation.

Sodium-glucose cotransporter 2 inhibitors (SGLT2i) are antidiabetic agents that demonstrate renoprotective effects in patients with diabetes. A critical appraisal of the existing evidence shows that their link to MAM regulation is largely indirect. Hosokawa et al. showed that ipragliflozin reduced BiP expression, alleviated ERS, and attenuated renal ELD in a diabetic model [135]. Similarly, Lee et al. reported that empagliflozin reversed high glucose-induced down-regulation of MFN2 and MFN1 (mitofusin 1) in HK-2 cells, thereby restoring key mitochondrial functions such as ROS regulation and ATP production [136].

Glucagon-like peptide-1 receptor agonists (GLP-1RA) have been shown to effectively slow the progression of renal injury in patients with CKD, particularly those with diabetes or obesity [137,125]. Existing evidence supports indirect links to MAM-related pathways: GLP-1RA activates AMPK signaling in diabetic kidneys, promoting FAO and inhibiting lipid synthesis, ultimately reducing renal lipid accumulation [138,139]. In addition, GLP-1RA suppresses the expression of ACSL4, a key enzyme involved in lipid peroxidation and ferroptosis, thereby protecting against lipid-induced renal injury [138,139]. Although direct evidence is limited, increasing attention has been paid to the potential involvement of MAM in mediating the renoprotective effects of GLP-1RA [140]. Supporting this possibility, GLP-1 has been reported to up-regulate MFN2 expression in vascular smooth muscle cells, which may enhance ER-mitochondria coupling [141].

To date, no studies have employed MAM-specific outcome measures, such as the quantification of ER–mitochondria contact sites, MAM proteomic profiling, or inter-organelle transport assays, to validate these proposed mechanisms in the kidneys. Further dedicated research is required to establish a direct causal link between these drugs, MAM function, and renal lipoprotection.

Collectively, targeting MAM to reverse lipid metabolic disturbances and related injuries in various kidney diseases represents a promising direction and may offer new opportunities for therapeutic development.

The potential treatment strategies are summarized below (Table 2).

**Table 2:** Potential therapeutic agents for lipid-related kidney damage associated with MAM

Types of drugs	Proteins, modulators, or drugs	MAM-related mechanisms	Nephropathy	Reference(s)
Potential agents	MFN2	Restore MAM integrity	AKI/DN	[100,142]
	DsbA-L	Up-regulate MFN2 and restore MAM integrity	DN	[128]
	4-PBA	Up-regulate MFN2 and restore MAM integrity	3-MCPD-induced AKI	[116]
	PACS-2	Restore MAM integrity	DN	[103,87]
	VX-11e (MAPK1 inhibitor)	Up-regulate PACS-2 expression and restore MAM integrity	DN	[66]
	RTN1A inhibitor	Inhibit RTN1A, disrupt interaction with VDAC1, and reduce MAM enrichment	DN	[14]
	STING-PROTAC (STING antagonist)	Inhibit STING-VDAC interaction, restore GRP75/VDAC coupling and MAM integrity	RCC	[88]
	Taurine (GRP75 inhibitor)	Down-regulate GRP75 and restore puromycin aminonucleoside-induced excessive ER-mitochondria proximity	NS	[89]
	HC-030031 (TRPA1 inhibitor)	Down-regulate GRP75 and reverse MAM enrichment	AKI	[120]
	Capsaicin (TRPV1 activator)	Activate AMPK signaling, down-regulate FUN14 domain containing 1, attenuate IP3R/GRP75/VDAC1 complex formation, and reduce MAM enrichment	DN	[130]
	CNPY2 inhibitor	Inhibit CNPY2, modulate PERK phosphorylation, and ameliorate MAM disruption	DN	[131]
Existing renoprotective drugs	Dioscin	Up-regulate MFN2 expression; alleviate oxidative stress and reduce lipid peroxidation	DN	[132]
	Rosuvastatin	Up-regulate GRP75 expression	-	[134]
	SGLT2i	Reverse high glucose-induced down-regulation of MFN1 and MFN2; suppress BiP expression in lipid-associated kidney disease	DN/CKD	[135,136]
	GLP-1 receptor agonists (GLP-1RA)	Up-regulate MFN2 expression to enhance ER-mitochondria coupling and stabilize MAM integrity; activate AMPK signaling to promote FA oxidation and inhibit lipid synthesis; suppress ACSL4-mediated lipid peroxidation	DN/CKD	[138,139,141]

## Discussion

**Figure 7:**
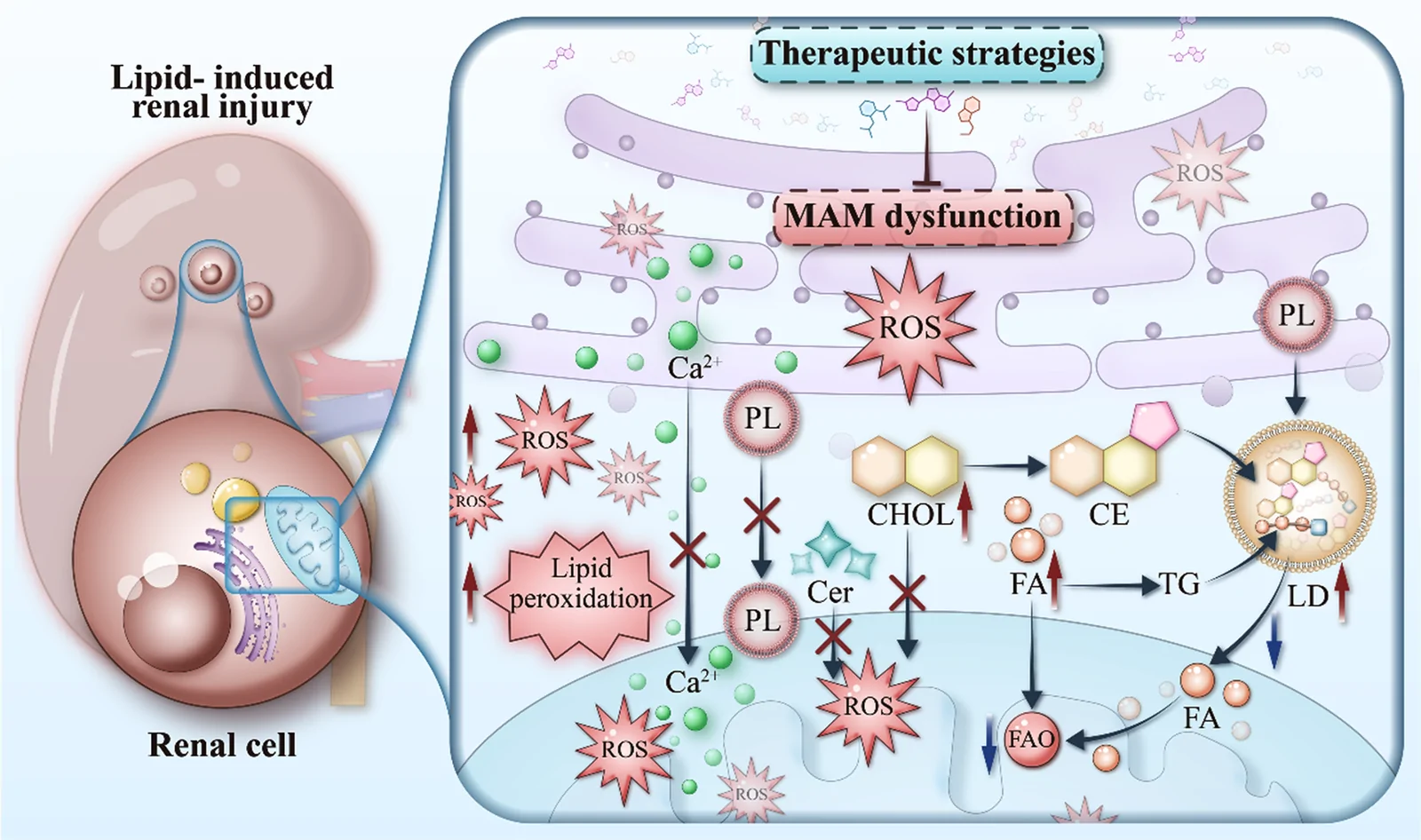
MAM-mediated Lipid Dysregulation in Renal Lipotoxicity Schematic overview showing that MAM dysfunction may perturb the metabolism of multiple lipid classes, including PL, CHOL, TG, FA, and ceramide (Cer), and may also disrupt LD homeostasis. In susceptible pathological contexts, these alterations can promote excessive ROS production, lipid peroxidation, ELD, and renal cell injury. Non-lipid stressors, including hyperglycemia, inflammation, hemodynamic stress, ischemia, and nephrotoxic insults, may independently induce renal injury or interact with MAM-mediated lipid dysregulation. Cer, ceramide; CHOL, cholesterol; FA, fatty acid; PL, phospholipid; ROS, reactive oxygen species; TG, triglyceride.

As the structural and functional interface between the ER and mitochondria, the MAM has attracted increasing attention. Although lipid metabolism was the earliest recognized function of the MAM [10,18], current studies on its regulatory role remain limited, and no dedicated review has systematically summarized existing findings on MAM-mediated lipid metabolism, especially in the kidney. Figure 7 provides a comprehensive overview of how the MAM coordinates diverse lipid metabolic pathways and how its dysfunction leads to renal lipotoxicity.

The present review focuses specifically on MAM lipid dysregulation in three metabolic kidney disease contexts with direct experimental support: diabetic kidney disease, obesity-related CKD, and metabolic stress-associated AKI [14,17,82,99,116]. We do not generalize these mechanisms to all forms of kidney disease. We stratify findings by model and evidence strength throughout—distinguishing human biopsy, *in vivo* rodent, *in vitro* renal cell, and non-renal extrapolations [14,17,74,78,82,90,91,99,116]. MAM-mediated lipotoxicity is a fundamental mechanism in the pathogenesis of metabolic kidney diseases. In contrast, non-metabolic kidney diseases, such as immune complex glomerulonephritis, obstructive nephropathy, polycystic kidney disease, and injuries associated with viruses or alcohol, have primary etiologies that are independent of MAM. While renal MAM research remains emerging, the present review synthesizes evidence from the past 3 years, including recent human biopsy data and clinical trial evidence of approved renoprotective drugs that modulate MAM function [17,125,134–140].

Compared with the nervous system and liver, research on MAM in kidney diseases remains relatively limited. Nevertheless, accumulating evidence indicates that MAM-associated proteins and regulatory mechanisms are largely conserved across tissues [72,91,117,124]. However, tissue-specific features exist. Structurally, the ER-mitochondria distance in renal tubular epithelial cells (5–20 nm) is comparable to that in hepatocytes but may be more dynamic in neurons [14]. Functionally, hepatic MAM is uniquely equipped for VLDL assembly and lipoprotein secretion, a pathway absent in renal epithelial cells [45]. Neuronal MAM is tightly linked to synaptic calcium signaling and amyloid precursor protein processing [22]. Proteomic analyses also reveal differential enrichment of certain MAM proteins. For instance, ACSL4 is highly expressed in both the kidney and brain but promotes ferroptosis in renal tubules while modulating synaptic plasticity in neurons [62,143]. These tissue-specific attributes underscore the need for kidney-focused investigations to fully understand the role of MAM in renal lipotoxicity.

Importantly, renal injury cannot be exclusively attributed to lipid dysregulation—hyperglycemia, altered hemodynamics, hypoxia/ischemia, inflammation, fibrosis, uremic toxins, and drug-related injury all contribute. Many forms of renal disease are not primarily driven by lipid abnormalities, with ELD often representing a secondary rather than primary pathogenic event. It is also critical to note that not all lipid disturbances cause renal diseases: CHOL and PLs are essential for membrane structure, FAs are the primary energy source for proximal tubular cells, ω-3 polyunsaturated fatty acid exert renoprotective effects, and LDs act as protective organelles that sequester cytotoxic FFAs. Renal damage is induced by particular lipotoxic species, such as saturated FFAs, Cer, oxidized LDL, and oxidized PLs, especially when oxidative stress and mitochondrial dysfunction are present. Merely accumulating lipids does not cause this damage.

Additionally, the present review focuses on MAM-mediated lipid regulation within renal cells and does not elaborate on extra-renal tissue contributions via inter-organ crosstalk. Adipose tissue dysfunction in obesity releases excessive FFAs, Cer, and proinflammatory factors that drive kidney injury [144]; MAM dysfunction in adipocytes also contributes to adipose tissue lipotoxicity and may indirectly promote renal lipotoxicity, though direct *in vivo* validation remains needed [145]; hepatic lipid dysregulation in metabolic dysfunction-associated fatty liver disease contributes to circulating atherogenic lipoproteins that accumulate in the kidney, forming a liver-kidney axis [146]. and gut microbiota dysbiosis generates lipotoxic metabolites that affect the kidney via the gut-kidney axis [147]. The contribution of extra-renal MAM dysfunction to kidney disease via inter-organ communication remains an important unanswered question and is beyond the scope of the present review [133,148].

**Figure 8:**
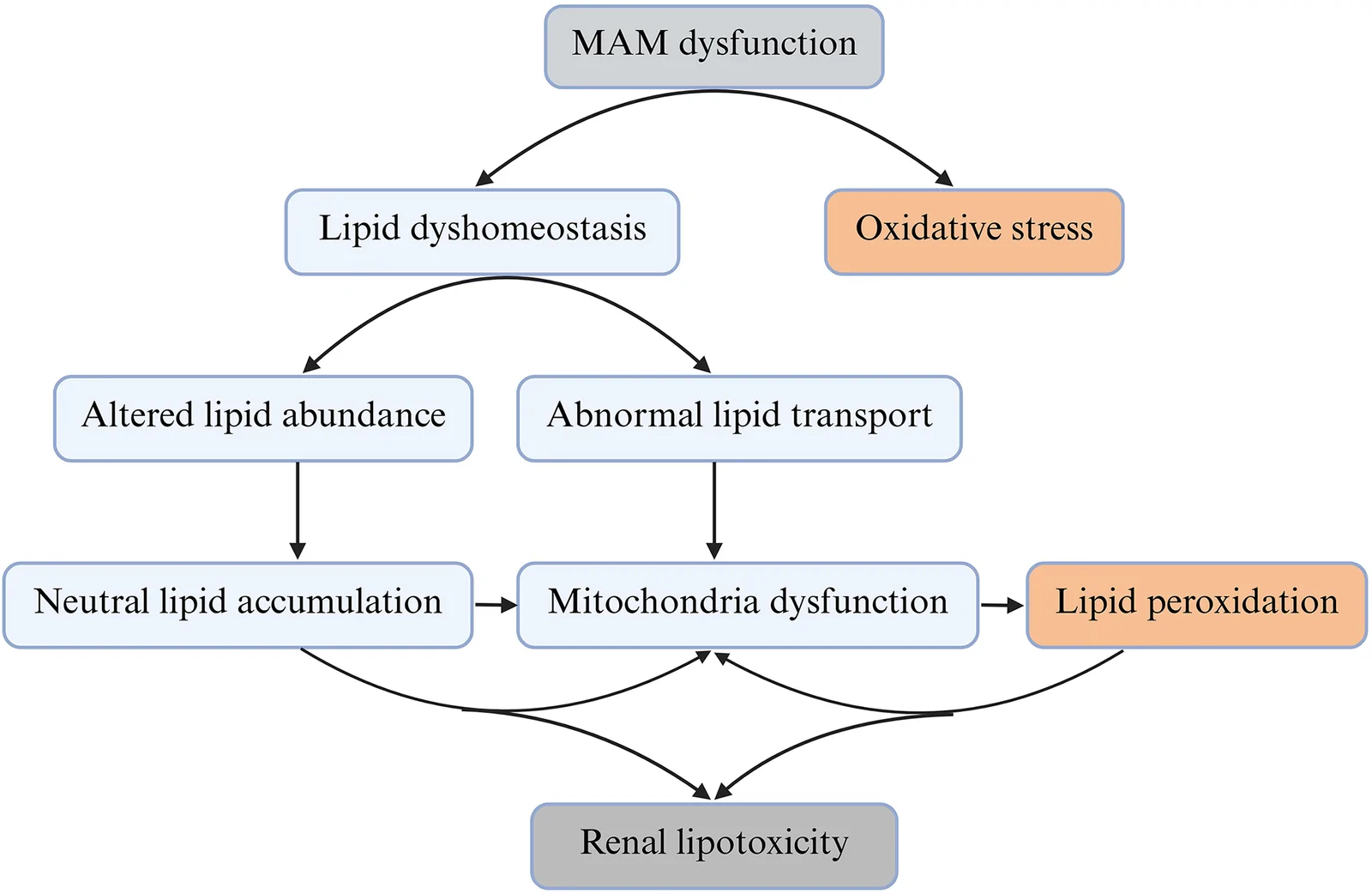
Proposed Mechanisms Linking MAM Dysfunction to Renal Lipotoxicity Proposed mechanisms linking MAM dysfunction to renal lipotoxicity. MAM dysfunction may (1) cause excessive accumulation of lipotoxic lipids; (2) promote abnormal deposition of neutral lipids as LDs; (3) induce redox imbalance and lipid metabolic dysregulation, which together promote lipid peroxidation; (4) impair mitochondrial function through multiple lipid-related mechanisms.

We frame the four-pathway model in Figure 8 as an integrative, testable hypothesis. The evidence levels are: (1) MAM-mediated PL transport and mitochondrial membrane maintenance are supported by direct evidence from *in vitro* renal tubular cell models and *in vivo* diabetic kidney disease models; (2) MAM-dependent ceramide trafficking and downstream mitochondrial apoptosis signaling have been validated *in vitro* in renal tubular cells and *in vivo* in AKI and renal fibrosis models; (3) MAM-mediated CHOL transport and mitochondrial free CHOL accumulation are supported by *in vitro* podocyte and tubular cell studies, with correlative evidence from human diabetic kidney disease biopsies; (4) MAM dysregulation leading to impaired FAO, LD accumulation, and lipid peroxidation/ferroptosis is supported by multiple independent *in vitro* and *in vivo* renal disease models. While each pathway has direct renal experimental support, the integrated model is a proposed framework, and validation of crosstalk and sequential interactions remains a future direction.

Directly linked to the present model is the MAM-ACSL4-ferroptosis axis. MAM represents the primary subcellular platform for ferroptosis execution, as it spatially couples the two required events for lipid peroxidation: the generation of peroxidation-susceptible polyunsaturated fatty acid-containing phospholipids (PUFA-PLs) and local production of ROS that drive lipid peroxidation. The core mediator of this axis is ACSL4, a confirmed MAM-localized enzyme that catalyzes the activation of polyunsaturated FAs for incorporation into membrane PLs, generating primary substrates for lethal lipid peroxidation during ferroptosis. In both AKI and diabetic kidney disease, MAM disruption leads to dysregulated ACSL4 localization and activity at the ER-mitochondria interface, driving excessive PUFA-PL accumulation and local lipid peroxidation in the immediate vicinity of mitochondrial membranes, which in turn triggers tubular ferroptosis and renal injury [97,98]. This axis is further supported by evidence that renoprotective agents, including GLP-1RAs, exert their anti-ferroptotic effects by preserving MAM integrity and normalizing ACSL4 activity at the MAM interface, providing a mechanistic link between MAM function, lipid metabolism, and regulated cell death in the kidneys [125,138]. This integrated MAM-ACSL4-ferroptosis pathway represents a key downstream mechanism by which MAM dysfunction drives renal lipotoxicity and connects the previously separate discussions of lipid metabolism and oxidative stress in the present review into a unified pathogenic cascade.

Regarding therapeutic translation, emerging preclinical and clinical evidence has identified multiple promising targets and approved drugs that modulate renal MAM function. The most translationally advanced agents are SGLT2i and GLP-1RA, with proven renal benefits in large-scale human trials. Mechanistically, SGLT2i up-regulate MFN2, restore ER-mitochondria coupling, and reduce ER stress in renal tubular cells [134,135]; GLP-1RA improve FAO, inhibit ACSL4-mediated ferroptosis, and preserve MAM integrity, with supporting evidence from both preclinical models and large-scale RCTs [125,136–140]. However, evidence directly linking these drugs to MAM modulation is entirely preclinical and indirect; no human trial to date has measured MAM-specific outcomes. Beyond approved drugs, preclinical targets validated in rodent models include up-regulation of MAM tethering proteins (MFN2, PACS2, and DsbA-L) [124,126–129], inhibition of pathological tethers (RTN1A and CNPY2) [14,130], and small-molecule compounds (TAU, dioscin, and TRPA1 inhibitors) [119,88,131]. A key limitation remains the complete absence of validated, MAM-specific biomarkers or clinical outcome measures, which represents the single largest barrier to clinical translation.

Finally, we offer our perspective on divergent findings regarding MAM enrichment versus disruption in kidney disease—some studies report increased MAM contacts while others observe reduced coupling [14,65,99,102]. One proposed explanation is that MAM contacts undergo dynamic, stage-specific regulation: in the early, acute phase, MAM may transiently increase as an adaptive compensatory response to support calcium homeostasis and lipid transport; in the late, chronic phase, prolonged stress leads to MAM disruption and irreversible dysfunction. The present model is supported by evidence from cardiac ischemia-reperfusion, liver non-alcoholic fatty liver disease/NASH models, and emerging renal ischemia-reperfusion and cisplatin-induced AKI models showing consistent time-dependent MAM changes [74,142]. However, this remains a testable hypothesis, and dedicated time-course studies across renal disease stages are required to fully resolve the paradox.

In conclusion, although the mechanisms by which MAM regulates lipid metabolism and redox homeostasis in the kidneys are not yet fully understood, accumulating evidence highlights its pathological relevance. MAM dysfunction contributes to renal ELD and lipid peroxidation, playing a pivotal role in lipid-induced renal injury. Targeting MAM holds promise as a therapeutic strategy, and further investigation into the mechanisms by which existing and novel agents modulate MAM function may provide valuable insights. Continued research in this area may ultimately facilitate the development of innovative strategies for preserving renal lipid homeostasis and improving clinical outcomes in patients with CKD.

## Abbreviations

3-MCPD: 3-chloro-1,2-propanediol
4-HNE: 4-hydroxynonenal
4-PBA: 4-phenylbutyric acid
5/6Nx: 5/6 nephrectomized
ABCA1: ATP-binding cassette subfamily A member 1
ABCG1: ATP-binding cassette subfamily G member 1
Acetyl-CoA: acetyl coenzyme A
ACSL: acyl-CoA synthetase long-chain family member
ACSL4: acyl-CoA synthetase long-chain family member 4
Acyl-CoA: acyl coenzyme A
AGPAT: 1-acylglycerol-3-phosphate O-acyltransferase
AKI: acute kidney injury
ALCAT1: acyl-CoA:lysocardiolipin acyltransferase-1
ALDH2: aldehyde dehydrogenase 2
AMPK: AMP-activated protein kinase
ApoB: apolipoprotein B
ApoE: apolipoprotein E
APP: amyloid precursor protein
AQP2: aquaporin-2
aSMase: acid sphingomyelinase
ATGL: adipose triglyceride lipase
ATP: adenosine triphosphate
BiP: binding immunoglobulin protein (also referred to as GRP78)
CACT: carnitine-acylcarnitine translocase
CE: cholesteryl ester
Cer: ceramide
CerS: ceramide synthase
CerS6: ceramide synthase 6
Ces1d: carboxylesterase 1d
CGL: congenital generalized lipodystrophy
CHOL: cholesterol
CKD: chronic kidney disease
CL: cardiolipin
CNPY2: canopy fibroblast growth factor signaling regulator 2
CoA: coenzyme A
CPT1: carnitine palmitoyltransferase 1
CPT2: carnitine palmitoyltransferase 2
DAG: diacylglycerol
DES: dihydroceramide desaturase (also referred to as DEGS)
DES1: dihydroceramide desaturase 1
DGAT2: diacylglycerol acyltransferase 2
DJ-1: Parkinsonism associated deglycase DJ-1 (also referred to as PARK7)
DN: diabetic nephropathy
DsbA-L: disulfide-bond A oxidoreductase-like protein
ELD: ectopic lipid deposition
EPT: ethanolamine phosphotransferase
ER: endoplasmic reticulum
ERLIN1/2: endoplasmic reticulum lipid raft-associated protein 1/2
ERO1α: endoplasmic reticulum oxidoreductin 1α
ERS: endoplasmic reticulum stress
FA: fatty acid
FAO: fatty acid oxidation
FFAs: free fatty acids
G3P: glycerol-3-phosphate
GLP-1: glucagon-like peptide-1
GLP-1RA: glucagon-like peptide-1 receptor agonists
GPAT: glycerol-3-phosphate acyltransferase
GPX8: glutathione peroxidase 8
GRP75: glucose-regulated protein 75 (also referred to as HSPA9)
GRP78: glucose-regulated protein 78 (also referred to as BiP)
HDL: high-density lipoprotein
HFD: high-fat diet
HMG-CoA: 3-hydroxy-3-methylglutaryl-coenzyme A
HMG-CoAR: 3-hydroxy-3-methylglutaryl-coenzyme A reductase
HSPA9: heat shock protein family A member 9 (also referred to as GRP75)
Insig-1: insulin-induced gene 1
IP3R: inositol 1,4,5-trisphosphate receptor
IRE1: inositol-requiring enzyme 1
LD: lipid droplet
LDL: low-density lipoprotein
LDLr: low-density lipoprotein receptor
LTPs: lipid transfer proteins
MAM: mitochondria-associated ER membrane
MAPK1: mitogen-activated protein kinase 1
MCSs: membrane contact sites
MDA: malondialdehyde
MFN1: mitofusin 1
MFN2: mitofusin 2
MIGA2: mitoguardin 2
MTP: microsomal triglyceride transfer protein
NLRP3: NOD-like receptor family pyrin domain-containing 3
NOX4: nicotinamide adenine dinucleotide phosphate oxidase 4
NS: nephrotic syndrome
nSMase: neutral sphingomyelinase
nSREBP2: nuclear sterol regulatory element-binding protein 2
OMM: outer mitochondrial membrane
ORP5: oxysterol-binding protein-related protein 5
ORP5/8: oxysterol-binding protein-related protein 5/8
ORP8: oxysterol-binding protein-related protein 8
PA: phosphatidic acid
PACS-2: phosphofurin acidic cluster sorting protein 2
PAP: phosphatidic acid phosphatase (also referred to as Lipin)
PC: phosphatidylcholine
PE: phosphatidylethanolamine
PEMT: phosphatidylethanolamine N-methyltransferase
PERK: PKR-like ER kinase
PL: phospholipid
PLA: proximity ligation assays
PLIN2: perilipin 2
PROTAC: proteolysis-targeting chimera
PS: phosphatidylserine
PSD1: phosphatidylserine decarboxylase 1
PSS: phosphatidylserine synthase
PSS1: phosphatidylserine synthase 1
PSS1/2: phosphatidylserine synthase 1/2
PSS2: phosphatidylserine synthase 2
PTIPIP51: protein tyrosine phosphatase-interacting protein 51 (also referred to as PTPIP51)
PTPIP51: phosphotyrosine interaction protein 51
PUFA: polyunsaturated fatty acid
PUFA-CoA: PUFA-coenzyme A conjugate
PUFA-PLs: polyunsaturated fatty acid-containing phospholipids
RCC: renal cell carcinoma
ROS: reactive oxygen species
RTN1A: reticulon-1A
SCAP: SREBP cleavage-activating protein
SEPN1: selenoprotein N
SERCA: sarco/endoplasmic reticulum Ca^2+^-ATPase
SERCA2: sarco/endoplasmic reticulum Ca^2+^-ATPase 2
SGLT2i: sodium-glucose cotransporter 2 inhibitors
Sig-1R: sigma-1 receptor
SM: sphingomyelin
SMase: sphingomyelinase
SPT: serine palmitoyltransferase
SPTLC1: serine palmitoyltransferase long chain base subunit 1
SPTLC1/2: serine palmitoyltransferase long chain base subunit 1/2
SPTLC2: serine palmitoyltransferase long chain base subunit 2
SQLE: squalene epoxidase
SQS: squalene synthase (also referred to as SS)
SREBP1/2: sterol regulatory element-binding protein 1/2
SREBP2: sterol regulatory element-binding protein 2
SREBPs: sterol regulatory element-binding proteins
StAR: steroidogenic acute regulatory protein (also referred to as STARD1)
StarD7: steroidogenic acute regulatory protein-related lipid transfer domain-containing protein 7
STING: stimulator of interferon genes
TAU: taurine
TGs: triglycerides
TMX: thioredoxin-related transmembrane protein
TRP: transient receptor potential
TRPA1: transient receptor potential ankyrin 1
TRPV1: transient receptor potential vanilloid 1
VDAC: voltage-dependent anion channel
VDAC1: voltage-dependent anion channel 1
VDAC2: voltage-dependent anion channel 2
VLDL: very-low-density lipoprotein
